# Epigenomic study identifies a novel mesenchyme homeobox2-GLI1 transcription axis involved in cancer drug resistance, overall survival and therapy prognosis in lung cancer patients

**DOI:** 10.18632/oncotarget.17715

**Published:** 2017-05-09

**Authors:** Leonel Armas-López, Patricia Piña-Sánchez, Oscar Arrieta, Enrique Guzman de Alba, Blanca Ortiz-Quintero, Patricio Santillán-Doherty, David C. Christiani, Joaquín Zúñiga, Federico Ávila-Moreno

**Affiliations:** ^1^ National University Autonomous of México (UNAM), Facultad de Estudios Superiores (FES) Iztacala, Biomedicine Research Unit (UBIMED), Lung Diseases And Cancer Epigenomics Laboratory, Mexico State, Mexico; ^2^ Instituto Mexicano del Seguro Social (IMSS), Centro Medico Nacional (CMN) Siglo XXI, Unidad de Investigación Médica en Enfermedades Oncológicas (UIMEO), Molecular Oncology Laboratory, Mexico City, Mexico; ^3^ National Cancer Institute (INCAN), Thoracic Oncology Clinic, Mexico City, Mexico; ^4^ National Institute of Respiratory Diseases (INER) “Ismael Cosío Villegas”, Mexico City, Mexico; ^5^ Harvard Medical School, Harvard School of Public Health, Department of Environmental Health, Boston, Massachusetts, USA

**Keywords:** lung cancer, homeobox transcription factors, epigenetics cancer drug resistance, overall survival, treatment prognosis

## Abstract

Several homeobox-related gene (HOX) transcription factors such as mesenchyme HOX-2 (MEOX2) have previously been associated with cancer drug resistance, malignant progression and/or clinical prognostic responses in lung cancer patients; however, the mechanisms involved in these responses have yet to be elucidated. Here, an epigenomic strategy was implemented to identify novel MEOX2 gene promoter transcription targets and propose a new molecular mechanism underlying lung cancer drug resistance and poor clinical prognosis. Chromatin immunoprecipitation (ChIP) assays derived from non-small cell lung carcinomas (NSCLC) hybridized on gene promoter tiling arrays and bioinformatics analyses were performed, and quantitative, functional and clinical validation were also carried out. We statistically identified a common profile consisting of 78 gene promoter targets, including Hedgehog-GLI1 gene promoter sequences (FDR≤0.1 and FDR≤0.2). The GLI-1 gene promoter region from −2,192 to −109 was occupied by MEOX2, accompanied by transcriptionally active RNA Pol II and was epigenetically linked to the active histones H3K27Ac and H3K4me3; these associations were quantitatively validated. Moreover, siRNA genetic silencing assays identified a MEOX2-GLI1 axis involved in cellular cytotoxic resistance to cisplatinum in a dose-dependent manner, as well as cellular migration and proliferation. Finally, Kaplan-Maier survival analyses identified significant MEOX2-dependent GLI-1 protein expression associated with clinical progression and poorer overall survival using an independent cohort of NSCLC patients undergoing platinum-based oncological therapy with both epidermal growth factor receptor (EGFR)-non-mutated and EGFR-mutated status. In conclusion, this is the first study to investigate epigenome-wide MEOX2-transcription factor occupation identifying a novel overexpressed MEOX2-GLI1 axis and its clinical association with platinum-based cancer drug resistance and EGFR-tyrosine kinase inhibitor (TKI)-based therapy responses in NSCLC patients.

## INTRODUCTION

Lung cancer remains the leading cause of death due to malignant disease in both the U.S.A. and worldwide, with 1.6 million cases annually [1, 2, 3, 4]. Non-small-cell lung carcinomas (NSCLCs) have been associated with exposure to carcinogenesis-promoting environmental risk factors, including tobacco smoke [5], as well several genetic and epigenetic aberrations in the lung [6]; however, notably, the number of lung malignancy-related deaths that are not tobacco-associated is continuously increasing [2, 6].

Several molecular mechanisms and cell-signaling pathways that may or may not be induced by exposure to environmental risk factors contribute to lung tumor biology, as reflected in the “Hallmarks of Cancer,” including genetic [8], transcriptional [9], and epigenetic aberrations in the cancer epigenome that are involved in [10–13] progression, survival and/or therapeutic responses in lung cancer patients [14–16].

Several studies have described epigenome-wide aberration profiles in lung cancer patients; some have identified DNA hypermethylation profiles at gene promoter sequences for transcription factors, while others have characterized the homeobox-related genes (HOX) *MSX1, OTX1, OSR1, IRX2, PAX6* [17], *SIX*, *LHX*, *PAX, DLX* and *ENGRAILED* [18], as well HOXA cluster genes such as *HOXA7* and *HOXA9* [19]. Notably, some HOX gene transcription factors have been proposed to be potential biomarkers for early diagnosis and/or to monitor treatment outcomes in lung cancer patients [20].

In NSCLC, certain mesenchyme-HOX (MEOX) family genes, such as MEOX2, have previously been associated with histopathological progression, poor clinical prognosis and oncological chemoresistance [21]. However, the mechanisms associated with HOX-related genes such as MEOX2 in the context of lung cancer drug resistance, overall survival, and clinical prognosis has yet to be fully elucidated.

In this study, we investigated the lung cancer epigenome to profile target gene promoters that are occupied and likely regulated by the transcription factor HOX-related gene MEOX2 accompanied by transcriptionally active RNA Pol II and epigenetic activation histone markers in human solid lung carcinomas. Bioinformatics analysis allowed us to identify a molecular signature consisting of 78 gene promoters (FDR≤0.2), particularly the Hedgehog-GLI1 gene promoter (with the stringent statistical values FDR≤0.1 and FDR≤0.2), accompanied by the enrichment of active RNA Pol II. In addition, quantitative validation and functional analyses confirmed that expression of the MEOX2-GLI1 transcriptional axis, accompanied by active RNA Pol II and the epigenetic activation histones H3K27Ac and H3K4me3, were involved in resistance to the cancer drug cisplatinum and lung cancer cell migration and proliferation. We also demonstrated that the MEOX2-GLI1 axis is clinically associated with poorer overall survival in lung cancer patients with both Epidermal Growth Factor Receptor (EGFR)-non-mutated and EGFR-mutated status. Thus, for the first time, we have described a novel mechanism by which the MEOX2-GLI1 axis is involved in lung cancer malignancy and platinum-based therapy resistance, as well as an association with clinical progression and poor overall survival, in both EGFR-non-mutated and EGFR-mutated lung cancer patients receiving EGFR-tyrosine kinase inhibitor (TKI)-based therapies.

## RESULTS

### MEOX2 immunoprecipitation on promoter tiling arrays reveals new MEOX2 gene promoter targets in lung cancer patients

**Figure 1:**
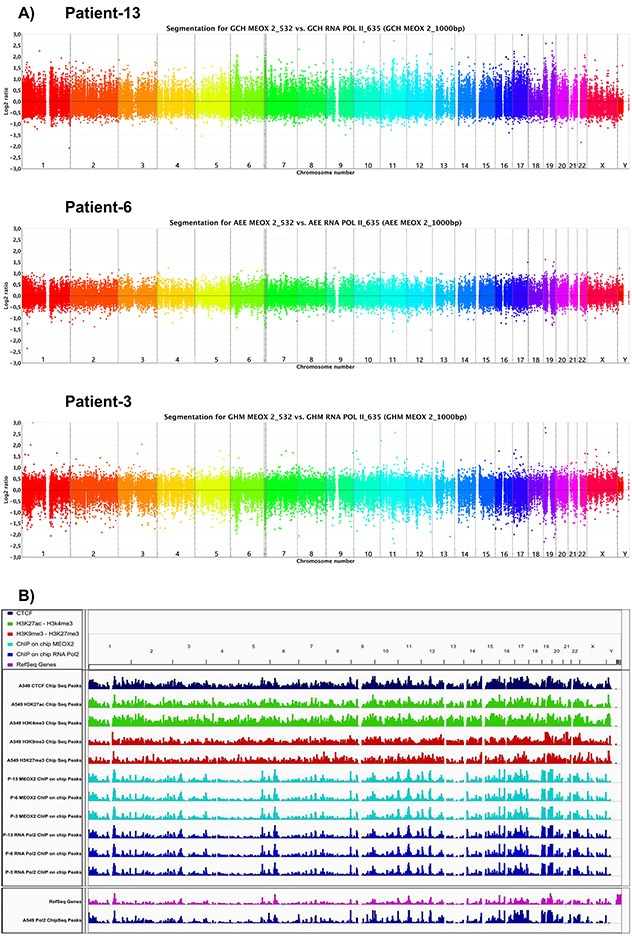
Bioinformatics analysis shows MEOX2 and RNA Pol II occupancy throughout the lung cancer epigenome to identify a new MEOX2 gene promoter target profile **(A)** Ideograms obtained via the CGH method indicate peaks of fluorescence intensity for channels 532/635, representing the enrichment of MEOX2_532 (above) and RNA Pol II_635 (bottom) throughout the lung cancer epigenome derived from lung adenocarcinoma patients. **(B)** Lung cancer epigenome, showing occupation of the CTCF insulator (dark-blue marks), activation histones H3K4me3 and H3K27Ac (green marks), and repression histones H3K9me3 and H3K27me3 (red marks) derived from A549 lung adenocarcinoma cells obtained from the ENCODE project database (deposited by the Broad Laboratory). Epigenome occupation by the transcription factor MEOX2 (turquoise marks) and RNA Pol II (dark-blue marks) in lung adenocarcinoma patients (generated by the present study). Additionally, the mRNA transcriptome profile (pink marks below) and RNA Pol II (dark-blue marks) epigenome occupation derived from A549 lung adenocarcinoma cells, taken from the ENCODE project database (deposited by the HudsonAlpha laboratory) are shown. All ENCODE tracks correspond to ChIPSeq assays, while present work tracks correspond to ChIP-on-chip assays utilizing gene promoter tiling arrays. All data were analyzed and visualized using IGV Viewer Program (version 2.3.60). Color codes have been included as a figure legend; increasing color intensity indicates the enrichment of epigenetic and transcriptional markers.

The primary goal of this study was to identify gene promoter targets of the HOX-related gene transcription factor MEOX2 in human solid lung carcinomas, to identify and propose new mechanisms, *e.g*., transcriptional axes, involved in lung oncological treatment resistance, and to characterize the association between clinical progression and overall survival in lung cancer patients. To address these questions, we implemented an epigenomics approach involving immunoprecipitation assays targeting MEOX2 and the RNA Pol II active enzyme using fragmented chromatin derived from 13 solid lung adenocarcinomas obtained from NSCLC patients ranging in age from 62 to 74 years, with clinical outcomes identified as lower (1 to 16 months) or higher (upper to 70 months) overall survival (Table I). The majority of cases demonstrated partial responses to first/second-line oncological treatment regimens based on cisplatinum/paclitaxel-navelbine and were selected to isolate immunoprecipitated DNA (IP-DNA) in MEOX2 and RNA Pol II immunoprecipitation assays that was used for subsequent competitive hybridization on NimbleGen promoter tiling sequence arrays, following a pipeline strategy descripted in Supplementary Figure 1A. Through bioinformatics analysis, we observed the enriched MEOX2 occupation of gene promoter sequences (Log2 above 532 nm fluorescence) *versus* RNA Pol II occupation (Log2 below 635 nm fluorescence) in the lung cancer patients designated P-13, P-6 and P-3 (Figure 1A). Differential MEOX2/RNA Pol II occupancy throughout the epigenome at gene promoter sequences was likely accompanied by the enrichment of active *versus* repressive epigenetic histone markers, as determined when we compared the epigenome of solid lung adenocarcinomas (identified in the present work) with the previously reported lung cancer epigenome from the lung cell line A549 obtained from the ENCODE project database (Figure 1B).

**Table I:** Clinical outcomes of the INER lung cancer patient cohort (n=13)

PATIENT	AGE	GENDER	WOOD SMOKE	TOBACCO	ASBESTOS/AROMATIC COMPOUND	NSCLC TYPE	TNM	FAMILY HERITAGE CANCER	FOLLOW UP	TREATMENT	CLINICAL RESPONSE	CURRENT STATUS	CANCER-DRUG FIRST LINE TREATMENT (CYCLES)	CANCER-DRUG SECOND LINE TREATMENT (CYCLES)
P-1	60	FEMALE	+	−	−	AD Solid	T2 N3 M1	+	94 months	Adjuvant Chemotherapy/Radiotherapy	Partial Response, Local Dissemination	ALIVE	CISPLATINUM/VINORELBINE Partial response (6)	ERLONITIB Partial response (6)
P-2	41	FEMALE	+	−	−	AD Solid	T3 N<3 M0	+	74 months	Coadjuvant and Adjuvant Chemotherapy/Radiotherapy	Partial Response	ALIVE	CDDP/NAVELBINE Partial response (6)	N.A.
P-3	62	FEMALE	+	−	−	AD Solid	T4 N3 M1	+	70 months	Adjuvant Chemotherapy/Radiotherapy	Partial Response, Progression, Local/Distal Dissemination	ALIVE	CISPLATINUM/NAVELBINE Partial response (6)	CARBOPLATINUM/PACLITAXEL Partial response (6)
P-4	77	FEMALE	+	−	−	AD Solid	T2 N<3M1	−	63 months	Adjuvant Chemotherapy/Radiotherapy	Response 75% reduction	ALIVE	CARBOPLATINUM/PACLITAXEL Partial response (6)	DOCETAXEL/ASA404 Partial response (6)
P-5	67	FEMALE	−	+	−	AD N.S.	T1 N<3M1	+	48 months	Adjuvant Chemotherapy	No Response, Local/Distal Dissemination	DEAD	CARBOPLATINUM/DOCETAXEL/BEVACIZUMAB Partial response (6)	PEMETREXED/CARBOPLATINUM No response (1)
P-6	70	FEMALE	+	−	-/+	AD N.S.	T4 N0 M0	−	16 months	Adjuvant Chemotherapy/Radiotherapy	Partial Response, Local/Distal Dissemination	DEAD	CARBOPLATINUM/GEMCITABINE No response (2)	N.A.
P-7	48	FEMALE	+	−	−	AD N.S.	T4 N<3M1	−	13 months	Adjuvant Chemotherapy	Partial Response	DEAD	CISPLATINUM/DOCETAXEL Partial response (6)	CARBOPLATINUM/GEMZAR No response (2)
P-8	71	FEMALE	−	+	−	AD N.S.	T4 N3 M1	−	11 months	Adjuvant Chemotherapy	No Response	DEAD	CARBOPLATINUM/DOCETAXEL Partial response (6)	CDDP/NAVELBINE PEMETREXED No response (1)
P-9	36	FEMALE	−	−	−	AD Solid	T4 N<3M1	−	11 months	Adjuvant Chemotherapy	No Response, Local/Distal Dissemination	DEAD	DOCETAXEL/CISPLATINUM Partial response (2)	PEMETREXED/CARBOPLATINUM No response (1)
P-10	68	FEMALE	−	−	N.S.	AD N.S.	N.S.	+	7 months	Adjuvant Chemotherapy/Radiotherapy	N.S.	DEAD	CARBOPLATINUM/PACLITAXEL Partial response (1)	ERLONITIB No response (1)
P-11	55	FEMALE	−	+	−	AD N.S.	N.S.	−	6 months	N.S.	N.S.	DEAD	N.A.	N.A.
P-12	64	FEMALE	+	−	−	AD N.S.	T4 N0 M0	−	2 months	Patient Refuse Treatment	Treatment Abandonment	DEAD	N.A.	N.A.
P-13	74	FEMALE	+	−	−	AD Solid	T3 N<3M1	−	1 month	Patient Refuse Treatment	N.A.	DEAD	N.A.	N.A.

Adenocarcinomas (AD). Not Specified (N.S.). Not Applicable (N.A.).

**Figure 2:**
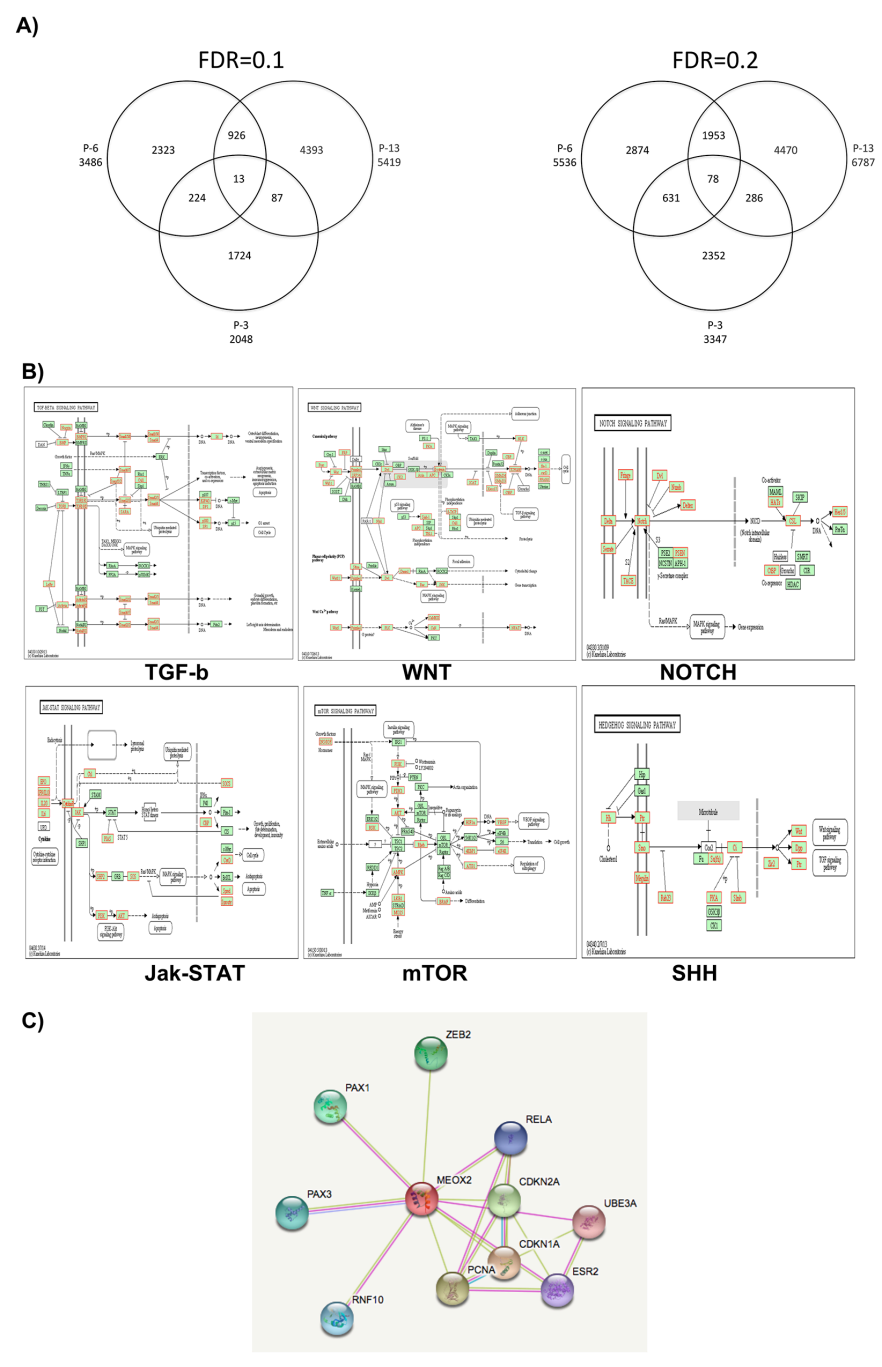
Cell signaling pathways predicted via bioinformatics analysis by the KYOTO genome expression database **(A)** MEOX2 gene promoter targets derived from lung cancer patients are clustered via Venn diagram analysis (patients P-13, P-6, and P-3). **(B)** Prediction analyses to identify cell signaling pathways, based on ChIP-on-chip bioinformatics data derived from MEOX2 gene target promoters in lung adenocarcinoma patients (representative image taken from P-6, AEE5536gensymbol.txt) with FDR≤0.2. AEE5536gensymbol.txt; organism: hsapiens; ID type: gene_symbol; ref set: genome, i.e., the entire Entrez Gene list; statistic: hypergeometric; significance level: 0.05; MTC: Bonferroni; minimum genes: 2. **(C)** Interactome protein-protein analysis predictions were performed based on selection of the MEOX2 protein using the STRING bioinformatics program (Version 10.0; http://string-db.org/cgi/network.pl?taskId=5WpBaIiJVSoV).

Additionally, bioinformatics analysis of 1,000-bp segments using the fluorescence index Log2-Ratio (MEOX2_532/RNA Pol II_635) identified a total of 6,340, 3,707 and 2,512 fluorescence peaks in the lung cancer patients P-13, P-6 and P-3, respectively, with statistical significance at FDR≤0.1 (Supplementary Figure 1B), representing a total of 5,419, 3,486 and 2,048 gene promoters in each patient, respectively (Figure 2A). Furthermore, 8,440, 5,577 and 4,317 fluorescence peaks were statistically significant at FDR≤0.2 (Supplementary Figure 1B), corresponding to a total of 6,787, 5,536 and 3,347 gene promoters in patients P-13, P-6 and P-3, respectively (Figure 2A).

Importantly, these findings suggest a statistically significant molecular signature consisting of 13 (FDR≤0.1) and/or 78 (FDR≤0.2) MEOX2 gene target promoters (Figure 2A; listed in Supplementary Table I) common to patients P-13, P-6 and P-3 despite their differing clinical survival data (Table I), likely controlling and/or modulating gene expression for cell signaling pathways.

### MEOX2 gene promoter targets are associated with cellular and embryonic differentiation signaling pathways in lung cancer patients

Using the MEOX2 gene promoter target data (Log2-Ratio MEOX2/RNA Pol II, FDR≤0.2), we predicted biological, molecular and cellular signaling pathways involved in embryonic development, protein-protein binding, ion binding, nucleic acid and nucleotide synthesis processes. Additionally, we investigated several cell-signaling pathways, such as TGF-b, WNT, NOTCH, MAPK, Jak-STAT, and mTOR, and Sonic Hedgehog (SHH) (Figure 2B), for which association with the Hedgehog-GLI1 gene promoter was statistically significant using both stringent FDR≤0.1 and FDR≤0.2 values (Figure 2A and Supplementary Table I). Interactome prediction analysis revealed a high probability of protein interactions among MEOX2 and protein nuclear factors such as the transcription factors PAX1, PAX3, RELA, and ZEB2, as well PCNA and CDKN1A-2A, which are involved in DNA replication and cell cycle control, respectively (Figure 2C).

### MEOX2 and RNA Pol II are positioned throughout GLI-1 gene promoter sequences

**Figure 3:**
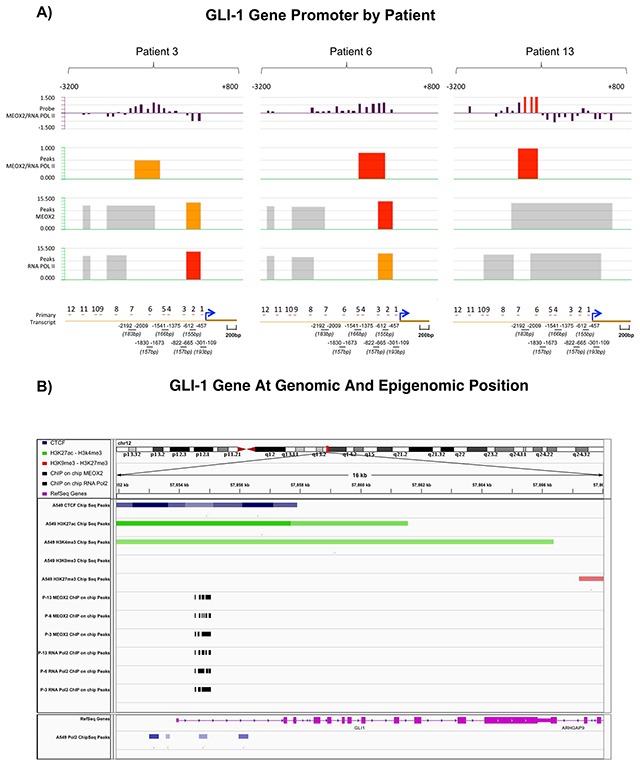
Statistically significant occupancy of Hedgehog-GLI1 gene promoter sequences by MEOX2 and RNA Pol II is accompanied by an activation histone profile in lung adenocarcinoma patients and lung cancer cells **(A)** Bioinformatics analyses identified 7 of 12 putative MEOX2-binding domains throughout GLI-1 gene promoter sequences (lower black bars) that were significantly associated with both MEOX2 and active RNA Pol II in lung adenocarcinoma patients (P-3, P-6, and P-13) and detected based on fluorescence peaks, which were determined via the alignment of at least 4 probes with a 90% or 95% call rate; statistically significant FDR values were obtained. **(B)** Bioinformatics analysis revealed CTCF insulators (dark-blue marks) as well as the histone activation markers H3K4me3 and H3K27Ac (green marks) and the absence of the repressive histones H3K9me3 and H3K27me3 (red marks) in A549 lung adenocarcinoma cells (ENCODE project database); this epigenetic activation pattern was accompanied by occupation of the transcription factor MEOX2 and active enzyme RNA Pol II (black marks) in solid lung adenocarcinomas (present study). Transcriptome expression profile (pink marks) accompanied by RNA Pol II (blue marks below) in A549 lung adenocarcinoma cells. All data were analyzed and visualized using the IGV Viewer Program (version 2.3.60). Color coding is provided in the figure legend; increased color intensity indicates the enrichment of epigenetic and transcriptional markers.

Bioinformatics analyses of GLI-1 gene promoter sequences between −3,200 bp and +800 bp were performed by evaluating the fluorescence absolute intensities obtained from individually imprinted probes and determining the fluorescence ratio of the *MEOX2_532 “upper”* and *RNA Pol II_635 “below”* channels (first track, Figure 3A).

Second-track (Figure 3A) fluorescence peaks were based on the fluorescence probe ratios (MEOX2/RNA Pol II Ratio) for the patients P-13 and P-6, which demonstrated fluorescence intensity peaks at FDR≤0.05 (red segments), while patient P-3 exhibited peaks (orange segments) at FDR≤0.1, primarily located from −3,200 to +1 bp in the GLI-1 gene promoter (Figure 3A).

Similarly, as shown in Figure 3A (third track), we identified MEOX2 fluorescence peaks in the GLI-1 gene promoter demonstrating a range of significance levels at FDR≤0.05, FDR≤0.1 and FDR≤0.2 (red, orange and gray color segments, respectively). Interestingly, in the fourth track, the significant presence of RNA Pol II on GLI-1 gene promoter sequences suggested that the GLI-1 gene is transcriptionally active, given the presence of the MEOX2 transcription factor, as well as epigenetically altered, demonstrated by a histone activation profile involving H3K4me3 and H3K27Ac and active enzyme RNA Pol II, which were previously identified in lung adenocarcinoma cells A549 obtained from the ENCODE project database analyzed here (Figure 3B).

### The GLI-1 gene promoter is occupied by MEOX2 and active RNA Pol II and is epigenetically associated with the activation histones H3K27Ac and H3K4me3

The bioinformatics results suggested the physical occupation and positive regulation of GLI-1 gene promoter sequences by the transcriptional factor MEOX2. To confirm this hypothesis, we quantitatively validated the MEOX2 occupancy of GLI-1 gene promoter sequences in 13 lung samples from patients with adenocarcinoma (Table I) and in the lung adenocarcinoma cell lines A427 and A549.

We designed an oligonucleotide set (Supplementary Table II) that covered 7 of 12 “TAATTA” MEOX putative transcription binding sites (spanning a sequence promoter region from −2,192 to −109 bp) identified via *in silico* prediction analysis using the TF-Search online program *Computational Biology Research Consortium* (see lower section of Figure 3A), and additionally we predicted 29 MEOX2 putative domains between −3,200 and −200 bp on the GLI-1 gene promoter using the bioinformatics program Jaspar (data not shown).

**Figure 4:**
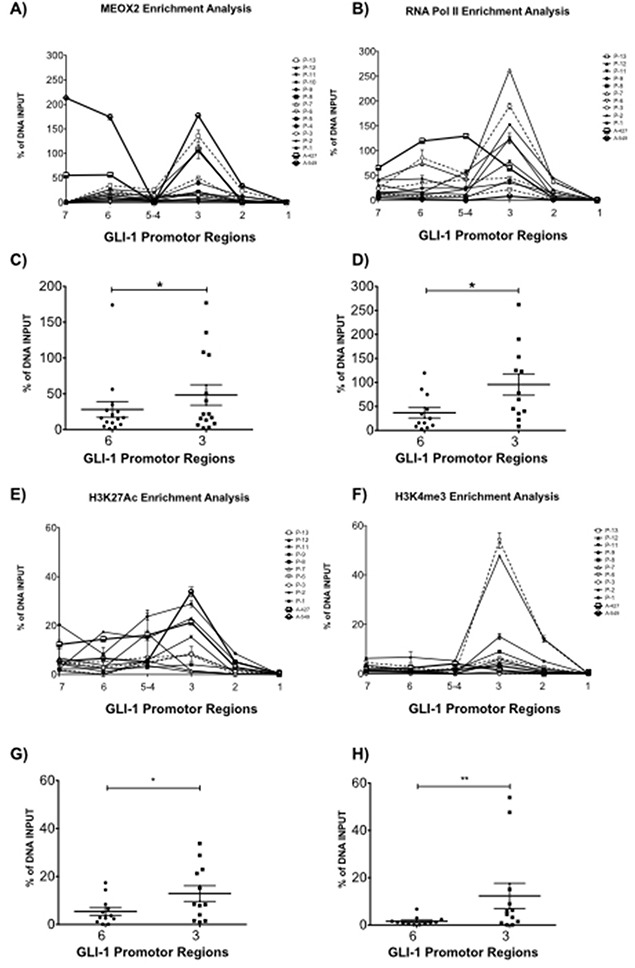
Quantitative validation analysis confirmed MEOX2 protein enrichment on GLI-1 gene promoter sequences accompanied by active RNA Pol II and the activation histones H3K4me3 and H3K27Ac **(A)** Quantitative enrichment analyses for MEOX2 and **(B)** RNA Pol II were performed on 7 of 12 putative MEOX2-binding domains in GLI-1 gene promoter sequences in samples from 13 lung adenocarcinoma patients as well as the A427 and A549 lung adenocarcinoma cell lines. Statistical analysis was performed to determine the average enrichment values for **(C)** MEOX2 and **(D)** RNA Pol II for each patient group, focusing on regions 6 and 3 located in GLI-1 gene promoter sequences. Histone code profile analysis was performed for **(E)** H3K27Ac and **(F)** H3K4me3 in 7 of 12 putative MEOX2-binding domains in GLI-1 gene promoter sequences, and the average enrichment was calculated, focusing on domains 6 and 3 for **(G)** H3K27Ac and **(H)** H3K4me3; *p≤0.05 indicates statistically significant differences (using a paired t test) between regions 6 and 3 in GLI-1 gene promoter sequences.

Quantitative qPCR analysis confirmed MEOX2 (Figure 4A) and RNA Pol II (Figure 4B) occupancy on GLI-1 gene promoter sequences in the region spanning −2,192 to −109 bp, and we detected higher levels of MEOX2 and RNA Pol II, particularly at MEOX2-putative 6 and 3 transcription binding sites (Figure 4A–4B).

MEOX2 and RNA Pol II occupancy on the GLI-1 gene promoter was differentially enriched, likely correlating with different clinical parameters such as overall survival time and/or cancer drug response (Table I). We identified significant (*p*≤0.05) quantitative enrichment in domain 3 compared to domain 6 of the GLI-1 gene promoter, including in the lung adenocarcinoma cell lines A427 and A549 (Figure 4C–4D). These findings indicate that MEOX2 occupancy is compatible with positive transcriptional modulation accompanied by enrichment of the epigenetic activation histones H3K27Ac and H3K4me3 (Figure 4E–4F), specifically the significant enrichment of H3K27Ac (Figure 4G) and H3K4me3 (Figure 4H) in domain 3 compared with 6 domain (*p*≤0.05), including in the A427 and A549 cell lines.

In contrast, decreased enrichment of the repressive histones H3K27me3 and H3K9me3 in both lung adenocarcinoma patients and the lung adenocarcinoma cell lines A427 and A549 was also detected on GLI-1 gene promoter (data not shown); instead an increased active histone profiling may also be confirmed for GLI-1 gene and promoter sequences in A549 lung cancer cells based on the bioinformatics analyses obtained from the ENCODE project database (Figure 3B). These results strongly suggest that positive transcriptional and epigenetic regulation of the MEOX2-GLI1 axis is involved in malignant lung tumor biology and cancer drug resistance *versus* responses, as previously suggested for MEOX2 in human lung cancer [21]; however, the mechanisms involved remain uncharacterized, and it is possible that a previously undescribed MEOX2 protein interaction on the GLI-1 gene promoter is responsible for lung cancer drug resistance and/or lung oncological therapy responses.

### Inducible mRNA and protein GLI-1 expression occur in a MEOX2- and cisplatinum dose-dependent manner in lung cancer

**Figure 5:**
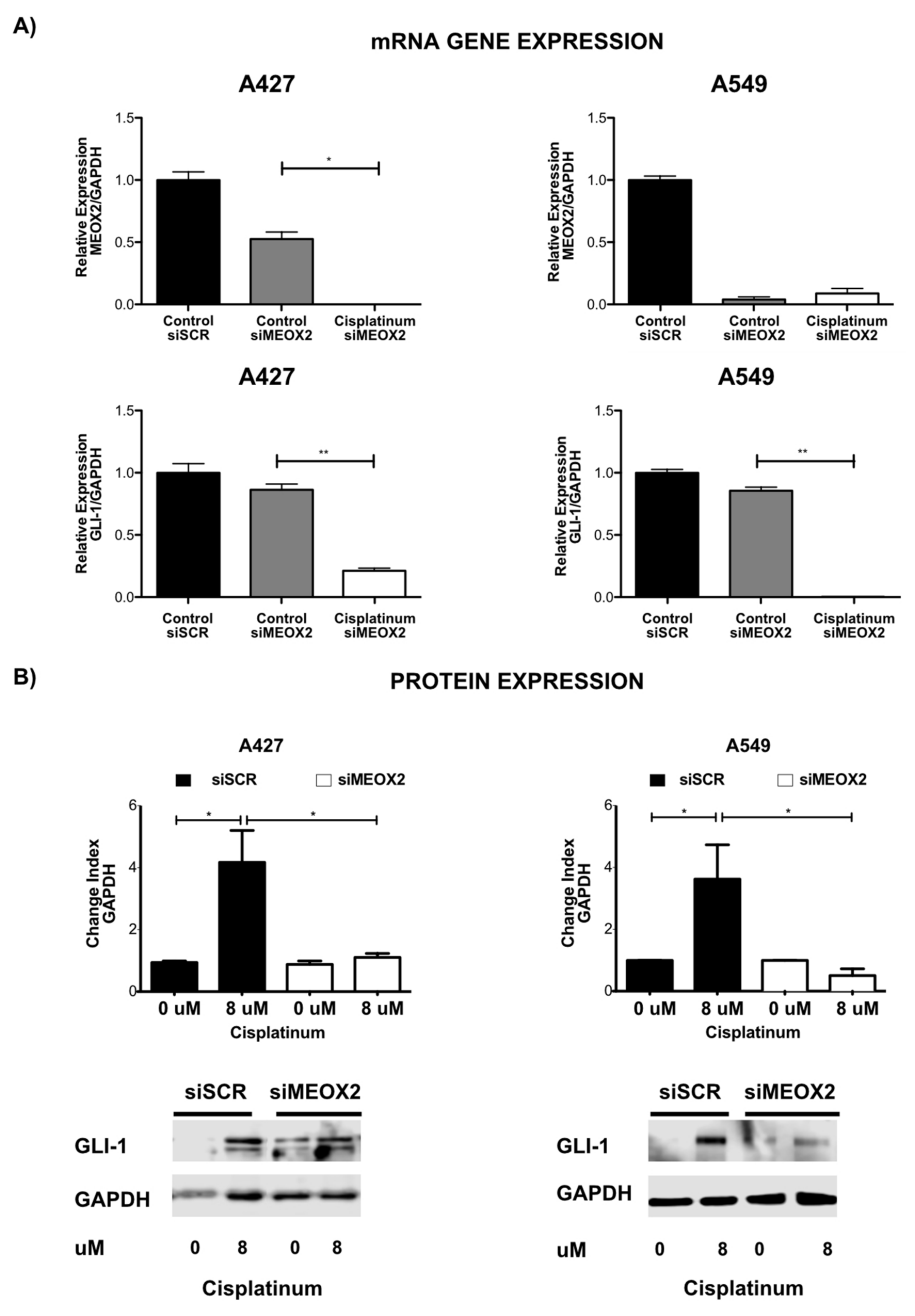
Inducible expression of GLI-1 mRNA and protein occurs in a MEOX2-dependent manner and is involved in cancer drug resistance **(A)** mRNA expression analysis was performed for the A427 and A549 lung adenocarcinoma cell lines in the presence or absence of cisplatinum-based treatment, and the effects of negative control scramble siRNAs and specific anti-MEOX2 siRNAs were assessed. The results are shown for two representative biological experiments performed in triplicate, with *p≤0.05, and **p≤0.01. **(B)** A427 and A549 lung adenocarcinoma cells exhibited a cisplatinum-inducible GLI-1 protein expression pattern at IC:12.5 (8 μM), while reduced inducible GLI-1 expression was observed following transfection with anti-MEOX2 siRNA in the presence of 8 μM cisplatinum. Western blot statistical analyses, assessed via quantitative densitometry, were performed to determine *p≤0.05 and **p≤0.005 using Student's *t*-test as well as one-way ANOVA with Dunnett's and Tukey's multiple comparison tests. Western blot bands were quantified as the pixel total intensity rate and expressed as a Change Index normalized to GAPDH. Images are representative of 3 biological replicates. Quantification analyses were performed using cisplatinum (0 μM) treatment as a negative control reference. Images were obtained using a C-DIGIT device (LICOR). Pixel quantification and data analyses were carried out using Image Studio software; the total pixel intensity for each specific protein product was normalized to GAPDH.

We observed a MEOX2-dependent reduction in GLI-1 mRNA expression both in the absence and the presence of cisplatinum and confirmed this observation using a specific anti-MEOX2 siRNA cocktail (Figure 5A). Specifically, reduced MEOX2 protein expression following the application of anti-MEOX2 siRNAs correlated with reduced GLI-1 protein co-expression levels (Supplementary Figure 2A).

Furthermore, an inducible, cisplatinum dose-dependent MEOX2 protein expression pattern was identified in lung adenocarcinoma cells (Supplementary Figure 2B), corresponding to cisplatinum-induced GLI-1 protein expression in the lung adenocarcinoma cell lines A427 and A549 that was markedly reduced by anti-MEOX2 siRNAs (Figure 5B).

**Figure 6:**
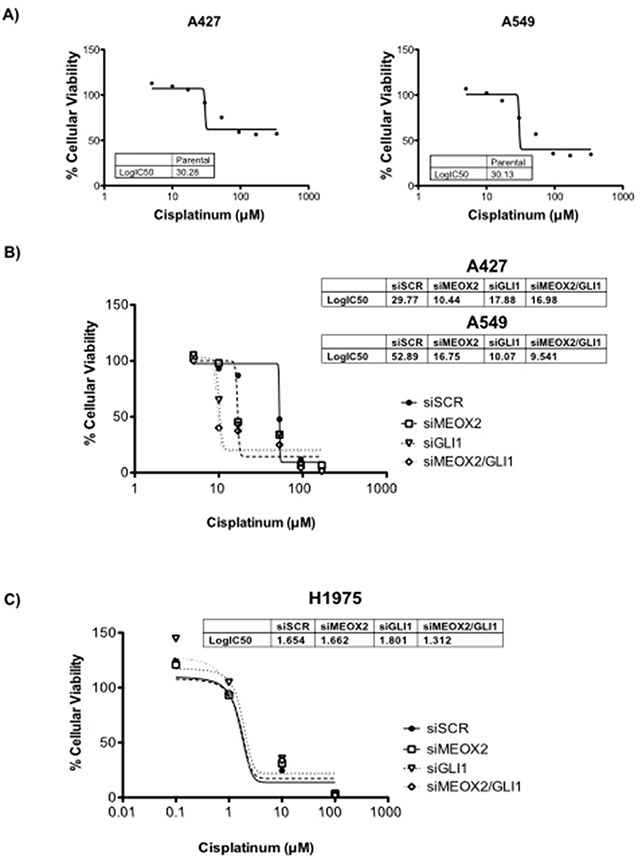
Cytotoxic cellular resistance exhibited MEOX2-GLI1 axis-dependent resistance via a cisplatinum dose-dependent mechanism **(A)** Cellular viability assays generated cisplatinum dose-response curves indicating the 50% inhibitory concentrations (IC:50) for A427 and A549 lung adenocarcinoma cells. **(B)** Cisplatinum IC:50s for both A427 and A549 lung cancer cells were significantly decreased (*p*≤0.05) by MEOX2 and/or GLI-1 gene silencing as determined by one-way ANOVA with Tukey's multiple comparison test. **(C)** Cisplatinum IC:50s for H1975 lung adenocarcinoma cells were not significantly decreased (*p*≤0.05) by MEOX2 and/or GLI-1 gene silencing.

To demonstrate the probable contribution of the MEOX2-GLI1 axis to chemoresistance, we performed cellular cytotoxic assays to evaluate cisplatinum dose responses using the lung adenocarcinoma cells lines A427 and A549. First, inhibitory concentrations (IC:50) were calculated by constructing cisplatinum dose-dependent curves; the IC:50 was approximately 30 μM for both cell lines (Figure 6A). Next, genetic silencing was performed using siRNA-MEOX2, and we observed a reduction in the IC:50s for both A427 (10.44 uM) and A549 (16.75 uM) cells. Similar effects were seen with siRNA-GLI1 or a mixture of siRNA-MEOX2 plus siRNA-GLI1 (Figure 6B), but no significant reduction in cellular viability occurred with either anti-MEOX2 or anti-GLI-1 siRNAs in an EGFR-mutated lung cancer cell line H1975 with non-detectable MEOX2 mRNA expression (Supplementary Figure 2C, Figure 6C). We concluded that MEOX2 transcription factor occupancy on the GLI-1 gene promoter acts as a positive modulator of MEOX2-GLI1 axis expression via a cisplatinum-dependent resistance mechanism associated with lung tumor cellular viability, cellular proliferation and migration.

### The inducible MEOX2-GLI-1 axis is involved in cellular migration and cellular proliferation in lung cancer cells

**Figure 7:**
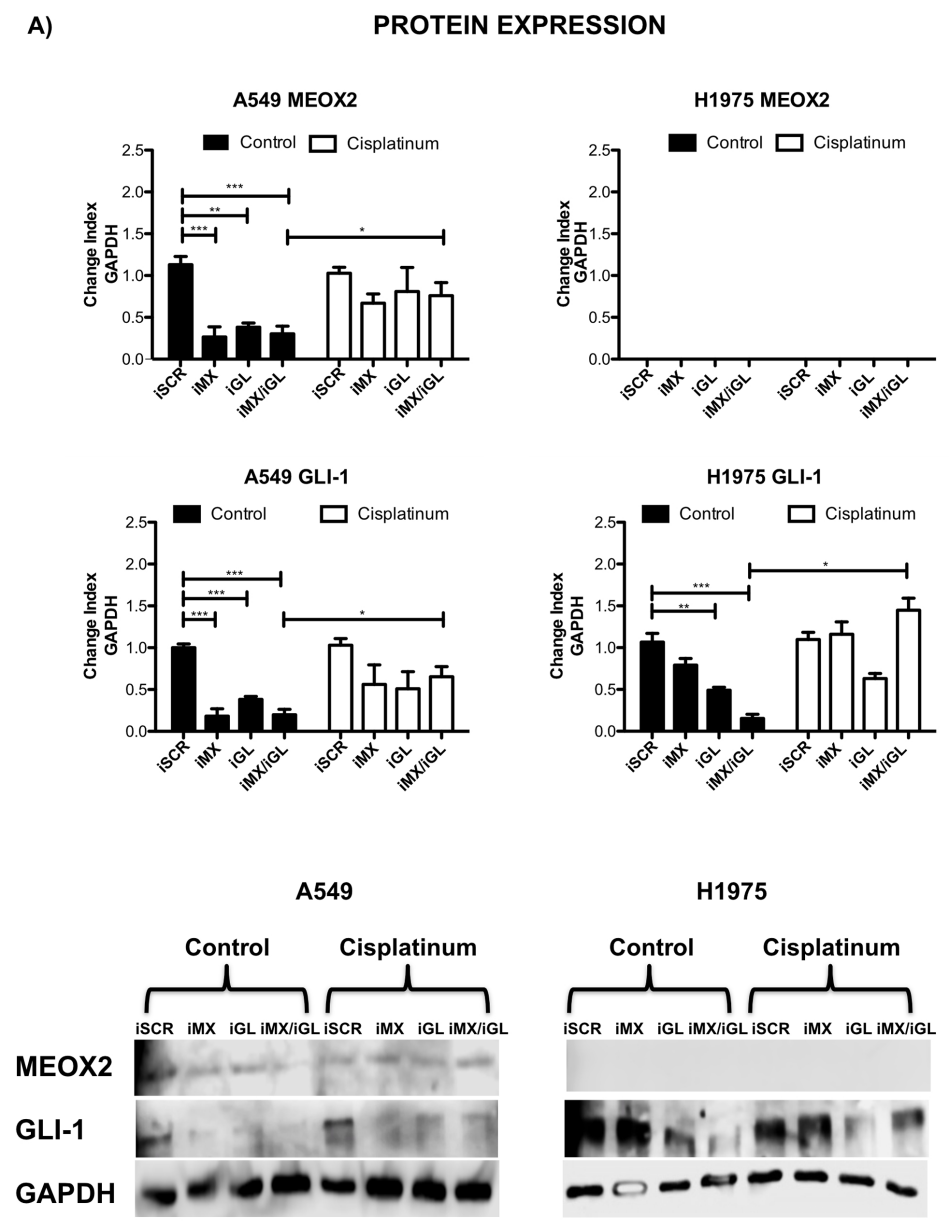

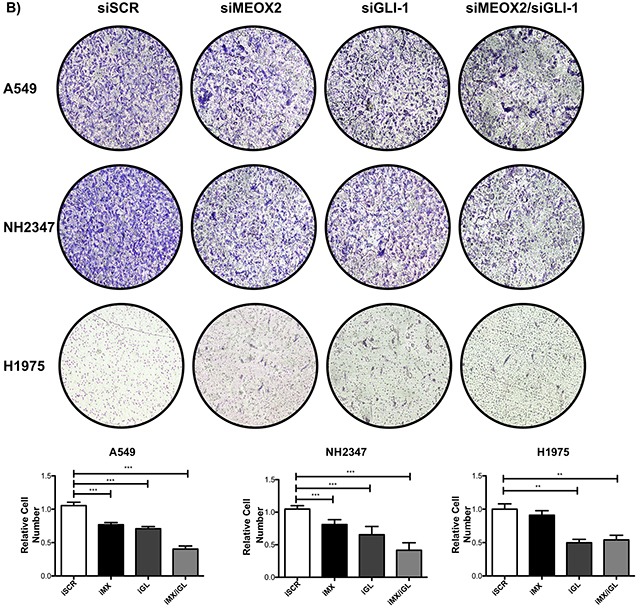

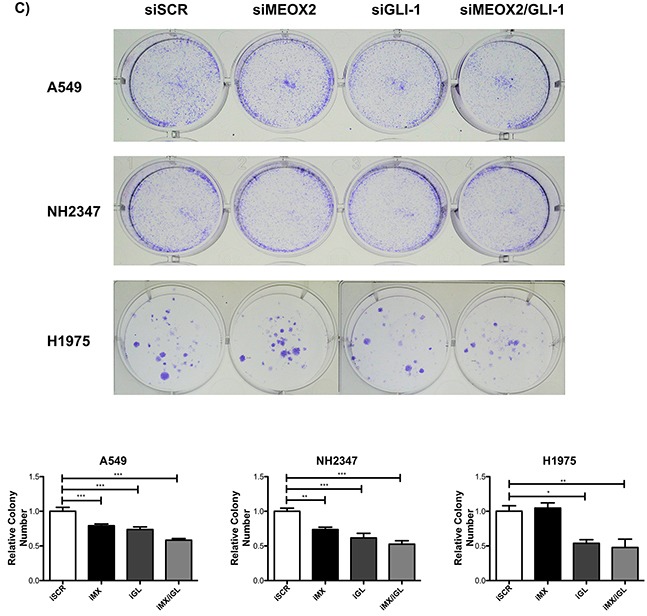
Inducible MEOX2-GLI1 axis expression was involved in cellular migration and cellular proliferation in lung cancer cells **(A)** A549 and H1975 lung cancer cells demonstrated an inducible GLI-1 protein expression pattern following treatment with 8 μM cisplatinum and reduced GLI-1 inducible expression following the application of specific anti-MEOX2 siRNA and/or anti-MEOX2 siRNA plus anti-GLI1 siRNA in the presence of 8 μM cisplatinum. Western blot statistical analyses, assessed via quantitative densitometry, were performed to determine *p≤0.05 by one-way ANOVA and Dunnett's test for multiple comparisons to identify significant differences with respect to controls. Student's *t*-test was performed to identify significant differences between control and cisplatinum treatment. Quantification analyses were normalized to scrambled siRNA as a negative control for gene silencing. Images were obtained using a C-DIGIT device (LICOR), and pixel quantification and data analyses were carried out using Image Studio software. Total pixel intensity for each specific protein product was normalized to GAPDH. **(B)** Cell culture images and graphs showing the quantitative analysis of cellular migration as a percentage (transwell migration assays) indicated significant MEOX2 and GLI-1 protein-dependent functions following the individual and combined application of anti-MEOX2 and anti-GLI1 siRNAs in A549, NH2347 and H1975 lung adenocarcinoma cells; **p≤0.005 and ***p≤0.0001 based on one-way ANOVA and Dunnett's multiple comparisons test. **(C)** Cell culture images and graphs showing the quantitative analysis of cellular proliferation (clonogenic assays) indicated significant MEOX2 and GLI-1 protein-dependent functions following the individual and combined application of anti-MEOX2 and anti-GLI1 siRNAs in A549, NH2347 and H1975 lung adenocarcinoma cells; **p≤0.005 and ***p≤0.0001 based on one-way ANOVA and Dunnett's multiple comparisons test. Transwell migration index and colony growth (clonogenic assays) rates were normalized and quantified using the ImageJ Colony Number plugin tool (see Materials and Methods).

Cellular migration and proliferation were systematically analyzed in functional assays, and significant (*p*≤0.001) MEOX2-dependent GLI-1 protein expression was present in A549 cells, which contrasted with non-detectable MEOX2 levels in the H1975 lung adenocarcinoma cell line (Figure 7A and Supplementary Figure 2C). Moreover, reductions in lung tumor cellular migration were observed in the A427 (Supplementary Figure 3A) and A549 (Supplementary Figure 3B) cell lines in an *in vitro* scratch-migration horizontal assay, and MEOX2 and/or GLI-1 gene silencing resulted in a significant reduction (*p*≤0.01 and *p*≤0.001) of the migration index in A549 cells (Supplementary Figure 3B), compared with a lower migration index in A427 cells (Supplementary Figure 3A). We also validated the role of the MEOX2-GLI1 axis by performing transwell-migration vertical assays with both the A549 and NH2347 lung adenocarcinoma cell lines and observed significant differences (*p*≤0.001) compared with H1975 lung cancer cells (Figure 7B), which express non-detectable MEOX2 levels (Figure 7A and Supplementary Figure 2C).

Finally, the MEOX2-GLI1 axis was validated by investigating the proliferation capacity of the A549 and NH2347 lung cancer cell lines in clonogenic assays, and we identified statistically significant differences (*p*≤0.01, and *p*≤0.001) compared with H1975 lung cancer cells (Figure 7C). All of these data are likely associated with clinical stage progression, malignant capacity (invasion/metastasis), and/or cancer drug resistance as well as overall survival in lung cancer patients.

### *In Situ* positive co-expression of MEOX2 and GLI-1 supports a MEOX2-GLI1 axis in two cohorts of lung cancer patients

Clinical validation analyses were performed in two independent lung cancer patient cohorts to identify positive nuclear co-expression of the MEOX2 and GLI-1 proteins *in situ* in solid lung carcinomas derived from 20 lung cancer patients in the “INER cohort” or 90 lung cancer patients in the “INCAN cohort” in the context of clinical outcomes (Table II), supporting our hypothesis regarding the existence of a novel co-regulatory axis involving association of the MEOX2 protein with GLI-1 gene promoter sequences in human NSCLC.

**Table 2:** Clinical outcomes of the INCAN lung cancer patient cohort (n=90)

	% (n/N)
**Gender**	
Female	63.3 (57/90)
Male	36.7 (33/90)
**Age (years)**	
Mean (± S.D.)	56.2 (14.1)
Median	58.5
**Median Age (years)**	
< 60	53.3 (48/90)
≥60	46.7 (42/90)
**Smoking Expossure**	
Non-smoker	48.9 (44/90)
Smoker	51.1 (46/90)
**Tobacco (pack/years)**	
Mean (± S.D.)	18.2 (22.9)
Median	10.3
**Median of Tobacco (pack/years)**	
Light smokers (< 10.18)	50 (23/46)
Heavy smokers (≥10.18)	50 (23/46)
**Wood-Smoke Exposure**	
Present	72 (65/90)
Absent	27.8 (25/90)
**Asbestos Exposure**	
Present	7.8 (7/90)
Absent	92.2 (83/90)
**Disease Stage**	
IIIb	23.3 (21/90)
IV	76.7 (69/90)
**TNM**	
TX	0(0/90)
T 0 to 1	0(0/90)
T2	12.2 (11/90)
T3	15.6 (14/90)
T4	72.2 (65/90)
NX	72.2 (65/90)
N0	0 (0/90)
N<3	0 (0/90)
N3	15.6 (14/90)
N4	12.2 (11/90)
MX	0 (0/90)
M0	23.3 (21/90)
M1	76.7 (69/90)
**ECOG PS**	
0-1	82.2 (74/90)
2-4.	17.8 (16/90)
**Histology**	
Adenocarcinoma	84.4 (76/90)
Epidermoid	7.8 (7/90)
Large Cell Carcinoma	5.6 (5/90)
Undifferentiated and others NSCLC	2.2 (2/90)
**Main Histological Pattern Among Adenocarcinoma Patients**	
Lepidic	14.5 (11/76)
Acinar	10.5 (8/76)
Papilar	7.9 (6/76)
Micropapilar	1.3 (1/76)
Solid	27.6 (21/76)
Other/Not specified	38.2 (29/76)
**Received Platinum-Based Chemotherapy**	
No	20 (18/90)
Yes	80 (72/90)
**Therapeutic Response to Platinum-Based Chemotherapy**	
Complete response	2.8 (2/72)
Partial response	40.3 (29/72)
Stable disease	40.3 (29/72)
Disease Progression	16.7 (12/72)
**EGFR-Mutated**	
Negative	60 (54/90)
Positive	36.7 (33/90)
Undeterminated	3.3 (3/90)

**Figure 8:**
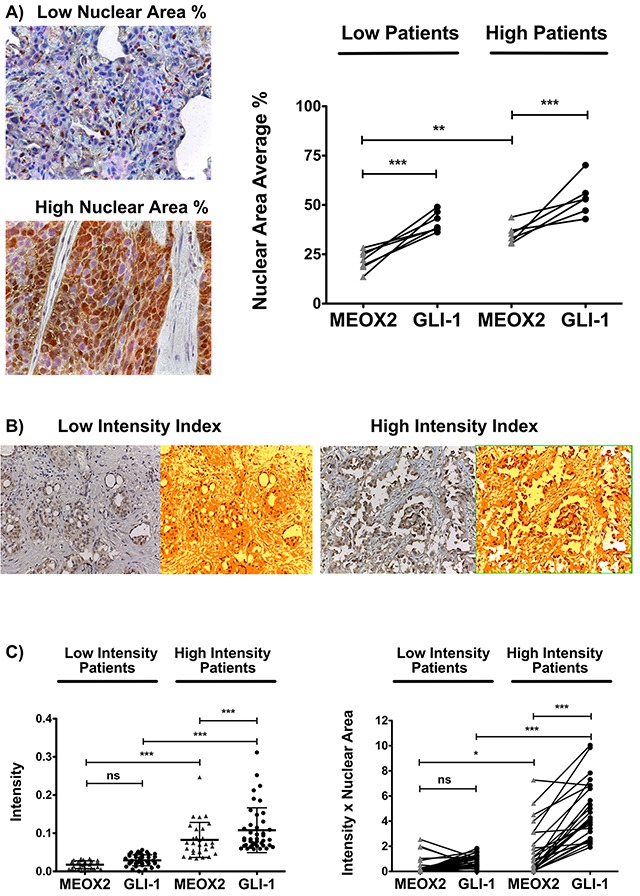
Positive MEOX2 and GLI-1 co-expression was confirmed in two lung cancer patient cohorts **(A)** Investigation of the lung cancer INER patient cohort by analyzing the expression intensity of MEOX2-DAB (brown) and GLI-1-AP (red) identified groups of lung cancer patients with lower and higher positive nuclear area averages (%) at **p≤0.005 and ***p≤0.0001. **(B)** Investigation of the lung cancer INCAN patient cohort by analyzing the intensity indices of MEOX2-DAB and GLI-1-DAB (not shown) via color transformation analysis based on the Algorithm Positive Pixel Count (see Materials and Methods) identified groups of lung cancer patients exhibiting lower and higher protein expression intensity levels with a cutoff established at *p≤0.05; significant differences were determined by performing one-way ANOVA and Dunnett's multiple comparisons test. **(C)** Intensity Index and Intensity Index plus Nuclear Area Average analyses for the independent positive expression markers MEOX2-DAB and GLI-1-DAB were performed, and groups of lung cancer patients exhibiting lower and higher protein expression levels were identified, with significant differences at *p≤0.05 and ***p≤0.0001 as determined by one-way ANOVA and Dunnett's multiple comparisons test.

In this context, double immunostaining analyses of cellular nuclear area expressed as an average percentage (MEOX2-DAB “brown” *versus* GLI1-AP “red”) were carried out for the INER patient cohort, and proportional positive co-expression levels for the MEOX2 and GLI-1 proteins (i.e., high MEOX2 expression levels correlate with high GLI-1 expression levels) were observed with *p*≤0.005 and *p*≤0.0001 significance levels, respectively (Figure 8A). Subsequently, this positive co-expression among both low and high protein expression lung cancer patient groups was quantitatively detected in the INCAN patient cohort by undertaking an Intensity Index analysis based on Algorithm Positive Pixel Bin Counts for MEOX2 and GLI-1 (Figure 8B). In addition, robust quantitative analyses were performed based on the Algorithm Intensity Index plus the average nuclear area percentage, confirming a positive correlation in terms of MEOX2-GLI1 axis co-expression levels for each lung cancer patient at both the *p*≤0.05 and *p*≤0.0001 significance levels (Figure 8C). Furthermore, based on clinical outcomes for the INCAN lung cancer patient cohort, which predominantly received platinum-based therapy (Table II), a multivariate analysis emphasized MEOX2 overexpression as a significant hazard risk, with a significance level of *p*=0.046 (Table III); these results support the existence of a role for the MEOX2-GLI1 axis in clinical malignancy and/or cancer drug-based therapy resistance *versus* response, reflected in overall survival.

**Table 3:** Multivariate analysis based on clinical outcomes of the INCAN lung cancer patient cohort (n=90)

	Relative Risk	95% C.I.	*P*
**Age**	1.013	0.99 – 1.04	0.709
**Gender**	1.145	0.53 – 2.5	0.73
**Smoking**	1.063	0.53 – 2.5	0.873
**ECOG**	1.227	0.82 – 1.8	0.323
**Clinical Stage**	1.138	0.74 – 1.7	0.354
**MEOX2 Expression**	2.04	1.02 – 4.1	0.046*
**GLI-1 Expression**	1.73	0.889 – 3.4	0.106

Cox regression model and relative risk (RR) ratio calculated with a corresponding 95% C.I.

### Overexpression of the MEOX2-GLI1 axis is associated with poorer overall survival and cancer drug therapy responses

**Figure 9:**
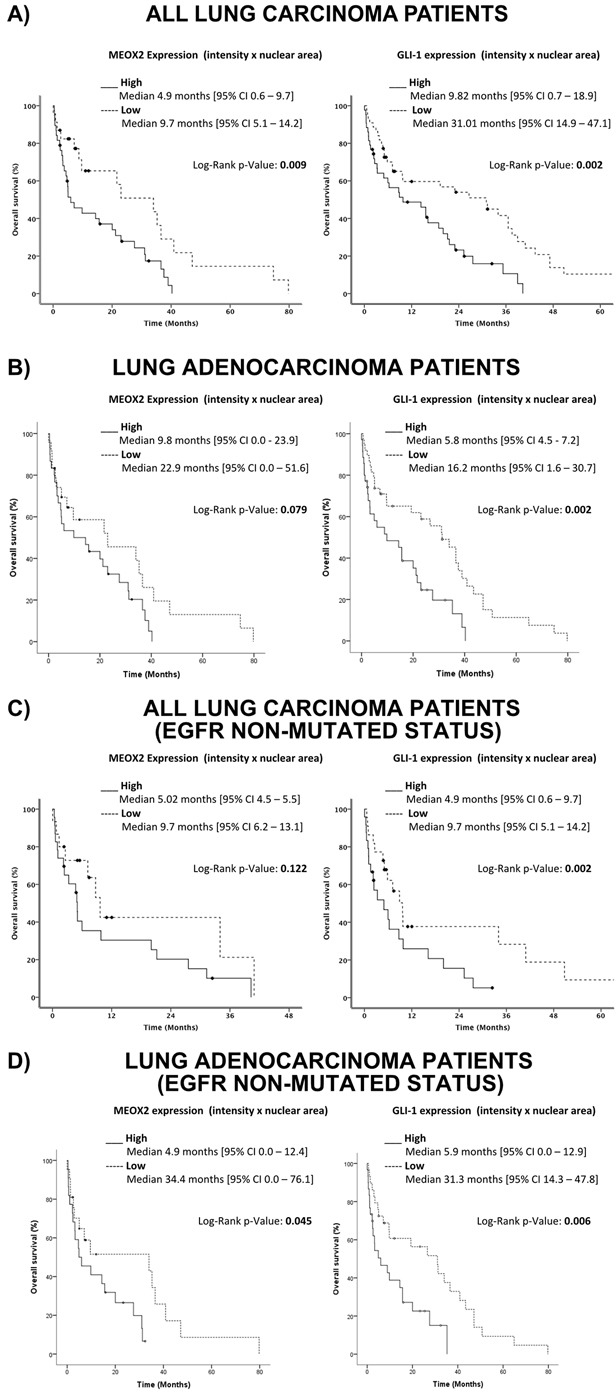
Quantitative (Intensity Index plus Nuclear Area Average) analyses revealed the involvement of the MEOX2-GLI1 axis in overall survival and clinical responses to treatment in lung cancer patients **(A)** Overall survival in all lung cancer patients with low and high MEOX2-GLI1 axis expression levels; global survival expressed as months accumulated (log rank “Mantel-Cox”, *p*=0.009 and *p*=0.002). **(B)** Overall survival in lung adenocarcinoma patients with low and high MEOX2-GLI1 axis expression; global survival expressed as months accumulated (log rank “Mantel-Cox”, *p*=0.079 and *p*=0.002). **(C)** Overall survival in all lung carcinoma patients with low and high MEOX2-GLI1 axis expression levels; global survival expressed as months accumulated under cancer drug treatment responses for EGFR non-mutated status (log rank “Mantel-Cox”, *p*=0.122 and *p*=0.002). **(D)** Overall survival in lung adenocarcinoma patients with low and high MEOX2-GLI1 axis expression Levels; global survival expressed as months accumulated under cancer drug treatment responses for EGFR non-mutated status (log rank “Mantel-Cox”, *p*=0.045 and 0.006).

Based on the analysis described above, a Kaplan-Maier survival curve analysis was constructed for the INCAN cohort, and better overall survival (statistically significant at *p*≤0.005) was observed for all lung carcinoma patients with lower MEOX2 and GLI-1 protein co-expression levels (statistically significant at *p*=0.009 and *p*=0.002, respectively) (Figure 9A). Additional Kaplan-Maier survival curve analyses of lung adenocarcinoma patients representing 84% (76/90) of the total INCAN lung cancer patient cohort indicated poorer overall survival (statistically significant at *p*≤0.005) in lung adenocarcinoma patients with higher MEOX2 and GLI-1 protein co-expression levels (*p*=0.079 and *p*=0.002, respectively) (Figure 9B).

Furthermore, survival curve analysis of all INCAN lung carcinoma patients with EGFR-non-mutated status identified a better overall survival index (statistically significant at *p*≤0.005) with lower MEOX2 and GLI-1 protein expression levels (*p*=0.122 and *p*=0.002, respectively) (Figure 9C). However, survival analysis of only lung adenocarcinoma patients with EGFR-non-mutated status identified poorer overall survival (statistically significant at *p*≤0.005) with higher MEOX2 and GLI-1 protein co-expression levels (*p*=0.045 and *p*=0.006, respectively) (Figure 9D).

Finally, overall survival analysis of all INCAN lung cancer patients with EGFR-mutated status identified better overall survival prognosis with lower MEOX2 and GLI1 protein co-expression levels (*p*=0.024 and *p*=0.011, respectively) (Supplementary Figure 4A-4B), which is likely associated with combined-cisplatinum plus TKI-based treatments for a specific group (33/90) of EGFR-mutated lung cancer patients (Table II). In summary, our results provide evidence for a novel transcriptional-epigenetic MEOX2-GLI1 axis that is involved in cellular viability, cancer drug resistance, cellular proliferation and migration capacity, and overall clinical survival and therapy prognosis in lung cancer.

## DISCUSSION

Genomic and epigenomic studies have described several genetic and epigenetic aberrations, some of which are identified as Homeobox-related gene transcription factors such as the HOXA and HOXB cluster genes, in human lung carcinomas [17, 18] that are involved in lung cancer biology [19] and are associated with early lung cancer diagnosis [20] and/or malignant stage upon clinical diagnosis [17]. Recently, MEOX2 has been implicated in fetal lung development and histopathological lung cancer progression [22], and MEOX2 and TWIST1 were previously associated with chemoresistance and lung cancer prognosis [21]. Recent published reports suggest MEOX2 overexpression accompanied by epigenetic events constitutes a probable new cancer drug resistance mechanism that must be considered in the context of lung cancer clinical management [23]. However, the identities of MEOX2 gene promoter targets, as well as related epigenetic profiles and molecular mechanisms involved in cancer drug resistance and clinical lung cancer prognosis, have remained unclear.

Here, we describe for the first time a MEOX2 gene promoter target profile in the context of transcriptionally active RNA Pol II enzyme in lung cancer. Our epigenome-wide data were directly obtained from human solid lung carcinomas and identified a range of 3,347 to 6,787 gene promoters (FDR≤0.2) representing several cell signaling pathways, including TGFb, WNT, Notch, Jak-STAT, MAPK, and mTOR; the involvement of some of these pathways in cancer progression, tumor cellular invasion and cancer metastasis has previously been described [24].

A statistically significant molecular signature consisting of 78 gene promoters (FDR≤0.2) or 13 gene promoters (FDR≤0.1) was shared by different lung carcinoma patients; these promoters included Sonic Hedgehog-GLI-1 gene promoter sequences, which have not been previously been reported to be under MEOX2 transcriptional influence. Importantly, the GLI-1 gene was recently proposed to be involved in ABC transporter-independent oncological resistance, in which the TWIST1 and Snail gene transcription factors are linked to the Sonic Hedgehog pathway and induce tumor-initiating cancer stem-like cells, in both non-solid tumor-derived leukemia cells and solid-epithelial tumor-derived cervical cancer cells [25]. Additionally, we suggest the MEOX2 transcription factor promotes platinum-based treatment resistance at the Hedgehog-GLI1 gene promoter level, in accordance with a previous report describing the involvement of Hedgehog-GLI1 in the ABCG2 transporter-dependent oncological resistance exhibited by a lung cancer stem-like side cell population [26] and is involved maintaining the self-renewal of cancer stem-like cells as recently reported for arsenic trioxide-treated lung carcinoma cells [27]. Here, we suggest not only the positive modulation of Hedgehog-GLI1 gene expression but also positive induction of the mTOR pathway (Figure 2), in line with very recent reports describing an association between the mTOR and 4E-BP1 pathways, which are under TWIST1 transcriptional control, and poorer overall survival in NSCLC patients [28], as well as the involvement of TWIST1-Snail, linked to the Hedgehog pathway, in a chemoresistant cancer stem cell-like phenotype [25].

The autocrine effects of the SHH pathway have previously been linked to cellular proliferation in NSCLC cell lines (A549, H322M, and HOP-62) and solid lung carcinomas derived from patients; specifically, GLI-1 genetic silencing combined with an SHH antagonist reduces lung tumor cellular proliferation [29]. Additionally, GLI-1 gene transcription factors have been functionally related to cancer stem cell self-renewal capacity and tumorigenicity in solid glioma tumors [30]. Furthermore, SHH-GLI1 signaling inhibition is involved in sensitization to EGFR-TKI (gefitinib/erlotinib)-based therapy, thereby reducing cellular viability, as well as the self-renewal function of cancer stem-like cells derived from the EGFR-mutated lung cancer cell lines H1650 and H1975 [31]. Here, we identified significant participation of the MEOX2-GLI1 axis in both cellular cytotoxic resistance capacity and cellular migration proliferation, which is likely explained in part by previous studies showing the involvement of the SHH-GLI1 signaling pathway in cellular proliferation, cellular transmigration and epithelial-mesenchymal transition in the NSCLC cell lines A549 and H520 [32]. In this regard, recent reports have demonstrated a correlation between MEOX2 overexpression and greater cellular migration capacity, increased senescence and cell cycle progression, promoting the development of vessel cells [33].

Several previous reports implicated the GLI-1 gene in docetaxel, methotrexate and etoposide chemoresistance through transcriptional modulation of the ABC transporter family genes in esophageal adenocarcinoma, prostate carcinoma and metastatic squamous cell carcinoma cell lines [34]. Other studies described the contribution of the canonical cell signaling SHH-GLI1 axis to acquired chemoresistance to doxorubicin, vincristine, and etoposide in both non-solid tumor-derived K-562 erythroid leukemia cells and solid tumor-derived epidermoid carcinoma KB cells [35]. These effects have also been observed in NSCLC cell lines under gefitinib/erlotinib exposure [31] and in mouse- and human-derived SCLC cells treated with the cancer drugs etoposide, carboplatin, cisplatinum and/or the SHH-antagonist NVP-LDE225 [36]. Additionally, a significant reduction in cisplatinum chemoresistance in experimental *in vitro* models is achieved via inhibition of the SHH-SMO-GLI1 axis signaling with an SMO antagonist (GDC-0449) in both human NSCLC and SCLC cell lines [37].

However, few studies have described chemoresistance at the transcriptional level in terms of gene promoter sequences. The ABCG2 transporter gene promoter is modulated by the GLI-1 gene transcription factor through gene sequences in other domains located from −416 to −408, accompanied by increased levels of the histone marker H3K18Ac, following cellular stimulation of the SHH-GLI1 axis [38]. Such epigenetic markers promote GLI-1 occupation of ABCG2 gene promoter sequences and induce the expression of ABCG2, which is involved in chemoresistance in the presence of doxorubicin and methotrexate in non-solid tumors and B-cell non-Hodgkin lymphoma cells [38]. These and other reports indicate the functionality of the SHH-GLI1-ABCG2 axis is highly correlated with chemoresistance, as shown for the lung cancer stem cell-like population derived from H460 parental lung cancer cells [39].

Our results obtained from solid lung carcinomas and the A427 and A549 NSCLC cell lines constitute the first evidence demonstrating significant epigenome occupancy of the homeobox-related gene MEOX2. Additionally, GLI-1 gene promoter sequences are transcriptionally occupied by active RNA Pol II and epigenetically accompanied by the active histones H3K27Ac and H3K4me3 with statistically significant enrichment at two hotspot regions ranging from −1,830 to −1673 and −822 to −665, supporting the existence of an active MEOX2-GLI1 axis, which was also indicated by the lung cancer epigenome analysis derived from A549 lung adenocarcinoma cells (Figure 1B and Figure 3B) previously published and deposited by Sabo PJ and colleagues in the ENCODE Project database [40].

Here, our findings are in accordance with intrinsic chemoresistance and/or also likely associated with acquired chemoresistance, which have been linked to and/or involved in epigenetic mechanisms based on aberrations in the histone repressive profiles H3K27me3 on MEOX2 gene promoter [21], but also histone activation profiles detected for H3K27Ac and H3K4me3 on GLI-1 gene promoter sequences (Figure 3B and Figure 4E-4H). But in other cases is modulated by long non-coding RNAs (lncRNAs), as was recently been reported for lncRNA HOTAIR, which promotes cisplatinum drug resistance by controlling p21 protein expression to increasing cell cycle control in A549 lung cancer cells [41]. Additionally, HOTAIR overexpression has been detected in solid tumor NSCLC patients with poorer prognosis as well as functionally associated with higher lung tumor cellular migration and cellular invasion *in vitro* lung in A549 and SPC-A1 cancer cells, with lung tumor metastasis *in vivo* in SPC-A1 lung cancer cells [42], and with poorer prognosis in lung cancer patients [37, 38]. Other possible explanations include a role for HOTAIR-coordinated assembly “binding” to the Polycomb Repressive Complex 2 (PRC2) in a SUZ12/EZH2 and/or LSD1/CoREST/REST complex-dependent manner, which are reportedly involved in increased repressive histone H3K27 methylation and H3K4 demethylation, respectively [43]. Additionally, lncRNA HOTAIR is epigenetically involved in repression of the p15, p21 and p27 genes at the promoter sequence level in lung cancer [44]. Down-regulated expression of lncRNA MEG3 is involved in cisplatinum resistance through p53 and Bcl-xl controlled gene expression in lung carcinoma cells [45], although additional epigenetic evidences must be obtained in lung cancer patients. Additionally, lncRNA BC087858 overexpression is clearly associated with acquired resistance to EGFR-TKI based “gefitinib” therapy [46], while lncRNA GAS5 down-regulation is involved in EGFR-TKI resistance in lung cancer patients and NSCLC cell lines [47]; in both cases, the underlying epigenetic mechanisms have yet to be fully clarified.

Our functional epigenomics strategy also suggests novel MEOX2-GLI1 transcriptional axis signaling is involved in resistance to the cancer drug cisplatinum in a dose-dependent manner, as previously proposed for lung cancer [21]. MEOX2 overexpression resulted in cisplatinum dose-dependent inducible GLI-1 gene expression at the gene promoter level associated with chemoresistance in lung cancer cells, which likely correlates with previous reports in which inhibition of the SHH-GLI1 axis is involved in cancer drug resistance by a lung cancer stem-like cell side population [37] through a direct transcriptional target action in the ABCG2 gene promoter sequence [38]. Additionally, this mechanism is involved in lung adenocarcinoma cell survival and cancer drug resistance conducted via the SHH-GLI1 axis but negatively regulated by Hedgehog-Interacting Protein (HHIP) in EGFR-non-mutated A549 or EGFR-mutated H1975 and HCC827 NSCLC cell lines under TKI-based treatment [48]; however, here this mechanism is positively regulated by MEOX2 at the transcriptional level.

In conclusion, here we have reported for the first time the functional and clinical correlations of the overexpressed MEOX2-GLI1 transcriptional axis, both as a novel molecular candidate axis involved in lung cancer drug resistance and as a predictor of therapy prognosis. Certain reports investigating NSCLC patients have described an overexpressed SHH-GLI2 axis associated with poorer progression-free survival [49] as well as an overexpressed GLI-2 axis involved in cellular proliferation and apoptosis resistance in lung squamous cell carcinomas [50]. However, as determined in SMO inhibition assays, these axes promote the down-regulation of GLI-1 gene expression and facilitate significant EGFR-TKI-based treatment susceptibility, thereby reducing tumor cell proliferation in both EGFR-wild type as well as EGFR-mutated NSCLC cell lines [51], suggesting an interdependent oncogenic addiction between the EGFR and SHH-GLI1 axis pathways that is involved in lung cancer cell survival and cancer drug resistance [52]. We obtained similar results for both EGFR-wild type and EGFR-mutated lung cancer patients (Figure 9C–9D and Supplementary Figure 4A). However a non-canonical GLI-1 activation likely occurs in a Rack1-dependent manner in NSCLC patients as a proposed necessary step for lung cancer tumorigenicity [53]. Additionally, this process is likely involved in EGRF-TKI-based therapy resistance in both EGFR-non mutated (A549) and EGFR-mutated (H1975) lung cancer cells [54].

Despite developing a significant correlation index based on the overexpressed MEOX2-GLI1 axis, our results depict a novel transcriptional and likely epigenetic mechanism involved in lung tumor biology and cancer drug resistance mechanisms to platinum-based and/or target EGFR-TKI-based therapy responses, affecting lung tumor cell viability, cellular migration/proliferation, clinical prognosis and overall survival in lung cancer patients.

## MATERIALS AND METHODS

### Sample selection and cell lines

A total of 123 tumor samples from 2 cohorts of NSCLC patients were included in the present study. The training cohort consisted of 13 fresh-frozen (FF) lung tumors collected from the Thoracic Surgical Service and 20 formalin-fixed paraffin-embedded (FFPE) lung tumors from the Pathology Service at the National Institute of Respiratory Diseases (INER) in Mexico City. An independent validation cohort consisted of 90 randomly selected patients with advanced NSCLC enrolled in the Thoracic Oncology Unit at the National Cancer Institute (INCAN) in Mexico City from July 2010 to December 2011. We included samples from patients with clinical stages corresponding to the TNM Staging System (6th edition) who had an Eastern Cooperative Oncology Group Scale of Performance Status (ECOG PS) score of 0–2 and were candidates for platinum-based chemotherapy and/or TKI-based target therapy.

In addition, the NSCLC cell lines A427, A549, NH2347 and H1975 from the American Type Culture Collection (ATCC, Manassas VA, USA) were used to perform experimental assays to investigate cisplatinum drug resistance and epigenetic analyses. The Institutional Review Board reviewed and approved protocols for INER (B17-07, B09-08) and INCAN (015/016/ICI, 964/15). All patients had a histologically confirmed lung cancer diagnosis, underwent surgical procedures, and provided informed consent before tumor resection for this study and future genetic/epigenetic biomarker studies.

### Clinical data collection, treatment regimen and patient follow-up

Clinical outcome data for all patients, including their clinical history, histopathological diagnosis, performance status and smoking history, were obtained from medical records. Smoking history was defined as ever smokers (combining current or former smokers). Current smokers were defined as those patients smoking who have smoked at least 100 cigarettes in life and were still smoking at the very moment of the interview. Former smokers were defined as those patients who were not smoking at the moment of the interview but have smoked at least 100 cigarettes in their life. All patients were treated according to international guidelines for lung cancer treatment [55]. All received platinum-based chemotherapy as a first-line treatment in combination with paclitaxel or vinorelbine. After progression with cytotoxic chemotherapy, the patients received second- or third-line treatment with an epidermal growth factor receptor (EGFR)-targeting tyrosine kinase inhibitor (TKI), either gefitinib or erlotinib, based on their mutation status. EGFR non-mutated and mutated patients (exons 18–21) were detected using a Therascreen RGQ EGFR Real-time PCR Kit (QIAGEN, Scorpions ARMS method, Dusseldorf, Germany), which was previously used to genotype NSCLC patients [56].

### Chromatin immunoprecipitation (ChIP)

FF lung tumor (LT; 3 mm^3^) samples consisting of almost 70% tumor tissue were pulverized under liquid nitrogen, fixed with 1% formaldehyde for 10 min, and immediately neutralized with 0.125 M glycine for 5 min. The resulting cells were washed with 1X PBS and treated with lysis buffer (50 mM HEPES-KOH pH 7.5, 140 mM NaCl, 1 mM EDTA pH 8, 1% Triton X-100, 0.1% sodium deoxycholate, and 0.1% SDS) containing 10 ml with protease inhibitors (complete Mini, Roche, Indianapolis, IN, USA).

The chromatin from the FF-LT samples was sonicated for 10 pulses of 20 seconds w/u 60 watts. Then, 10 μg of chromatin was immunoprecipitated (ChIP) using a commercial EZ-Magna ChIP™ G kit (Millipore, Temecula, CA, USA) and 2.5 μg of anti-MEOX2 (Santa Cruz Biotechnology, Dallas, TX, USA) as well as 1 μg of activated anti-H3K27Ac, anti-H3K4me3, anti-H3K27me3, anti-H3K9me3 or anti-RNA Pol II antibodies (Abcam, Cambridge Science Park, Cambridge, U.K.). A 1-μg aliquot of anti-mouse IgG was used as a negative control for ChIP (Millipore, Temecula, CA, USA).

### IP-DNA amplification, labeling and hybridization using promoter sequence tiling arrays

Sequence promoter tiling arrays (RefSeq, NimbleGen Assembly HG18, version 3×720K. Roche NimbleGen, Germany) representing 30,893 transcripts, 22,542 promoter sequences, and enhancer regions were employed to identify binding sites for the MEOX2 transcription factor. Microarrays containing 50-75-bp probes with 100 bp between each probe, representing −3,200 bp upstream and +800 bp downstream of the transcription start site (TSS), were used.

Immunoprecipitated DNA (IP-DNA; 20 ng) was linearly amplified using a Whole Genome Amplification (WGA2) GenomePlex Kit (IP-DNA-WGA2; 500 ng) and Reamplification Kit (WGA3) (Sigma, St. Louis, MO, U.S.A.). In both cases, WGA2 and WGA3 were amplified under the following conditions: Initial denaturation step at 95°C for 3 min, 14 cycles of denaturation/annealing/extension at 94°C for 15 seconds, and a final step at 65°C for 5 min. Re-amplified IP-DNA was purified using the phenol-chloroform method.

Next, a commercial NimbleGen Dual-Color DNA Labeling Kit (Roche, Germany) was used to label 1 μg of IP-DNA obtained with anti-RNA Pol II and anti-MEOX2 via incubation with a solution of nonamers (Cy3 and Cy5, respectively), 10 mM dNTP mix and 50 U/μl exo-Klenow fragment (3’-> 5’ exo-) for 3 hours at 37°C in the dark. Finally, 10 μl of buffer (0.5 M EDTA) was added to stop the fluorescence labeling reaction.

Total labeled IP-DNA was purified and washed with both absolute isopropanol and 70% ethanol and quantified at 260 nm using a spectrophotometer (Bioteck, model EPOCH, USA). Then, 34 μg of previously labeled IP-DNA obtained using both the MEOX2 and RNA Pol II antibodies was mixed and hybridized for 48 hours at 42°C. Finally, the microarrays were washed using the NimbleGen Wash Buffer kit (Roche Germany) and subjected to dry centrifugation (Roche NimbleGen, Germany) for 2 min at 1,500 rpm, and images were captured.

### ChIP-on-chip bioinformatic analysis

Microarray images were obtained at 532 nm and 635 nm using a scanner (GenePix model 4000a, USA). In addition, bioinformatics analysis was performed using NimbleScan software version 2.6 (NimbleGen, Germany).

Both images (one from each channel) were subjected to microarray alignment with values close to 0.1290, adjusting for brightness and contrast, and stored using the .tiff format. Next, ChIP-on-chip analysis generated the following bioinformatics reports containing information from both fluorescence channels: Pairs reports (.pair), Probe reports (.probe) and GFF reports (General Feature Format: .gff).

Additionally, a complete genome bioinformatics sub-analysis was performed using the CGH method to show the presence or absence of the fluorescence signals obtained from IP-DNA segments attached to MEOX2 and/or RNA Pol II, indicating possible transcriptionally active MEOX2 gene promoter sequence targets. Later, NimbleGen SignalMap (software version 1.9) was used to map the positions of the signal segments resulting from the hybridization data; for this analysis, a Log_2_-Ratio scale was established, and the index (ratio) was obtained using the formula 532/635 (*MEOX2_532/RNA Pol II_635*). Peaks were detected based on fluorescence intensity and determined by the alignment of 4 probes near one another (min probe > cutoff “cutoff peak”), contained in a sliding window of 500 bp.

While the cutoff value was evaluated based on a hypothetical 95 maximum percentage defined as the mean+6 [standard deviation], data from the index (ratio) were randomly assessed 20 times to establish the probability of a positive false discovery rate (FDR); the values FDR≤0.05, FDR≤0.1 and FDR≤0.2 indicate fluorescence peaks. (all epigenome data associated have been deposited in the online Dryad Digital Repository System, with the provisional doi:10.5061/dryad.rm7dd).

### Signaling prediction pathways (from MEOX2 gene promoter targets)

Hierarchical data analyses based on FDR probability and the *in silico* prediction of gene expression levels identified and selected genes that were most likely to be under the transcriptional control of MEOX2 in lung cancer patients. These analyses to predict metabolic pathways or signaling pathways were undertaken using the online KEGG (Kyoto Encyclopedia of Genes and Genomes) database, and *in silico* prediction analysis was performed using the online webpage http://www.webgestalt.org with the following statistical parameters: a hyper-geometric method and Bonferroni correction tests with a significance level of 0.05, and a minimum number of 2 genes per category.

### qPCR assays to quantitatively validate ChIP analyses

The IP-DNAs were analyzed by performing qPCR absolute quantification using a LightCycler 480 System (Roche, Mannheim, Germany), SYBR Green Master Mix (KAPA Science, Foster City, CA, USA) and oligonucleotide sets for the proposed gene promoter target sites (Supplementary Table II).

A total of 20 ng of IP-DNA was used per reaction, followed by the amplification of standard curves through serial dilutions of native DNA (diploid control genomic DNA) derived from the peripheral blood mononuclear cells of healthy donors (100 ng, 10 ng, 1.0 ng, 0.1 ng and 0.01 ng).

The following amplification conditions were used: initial denaturation at 95°C for 10 min and 40 cycles of denaturation at 95°C for 15 seconds, annealing at 55°C for 30 seconds and extension at 72°C for 30 seconds. Oligonucleotides (Supplementary Table II) were designed using the OLIGO and NT VECTOR programs and synthesized by SIGMA-ALDRICH (USA).

### Genetic silencing (siRNA transfection assays)

We used the anti-MEOX2 (sc-106233), and GLI-1 (sc-37911) siRNAs, each consisting of a pool (cocktail) of 3 target-specific siRNAs (19-25 nt) designed to knock down corresponding human gene expression. Two non-human related siRNAs obtained from Santa Cruz Biotechnology (Dallas, TX, USA) were used as negative controls (sc-37007 and sc-44230). Briefly, 3×10^5^ cells in RPMI-1640 (Biowest, USA) and 3% antibiotic-free FBS (Biowest, USA) were incubated in 6-well plates for 12 hours and then transfected for a total of 48 hours with Lipofectamine 2000® (Invitrogen, Foster City, CA, USA) and siRNAs at 50 nM in the presence or absence of a cisplatinum drug in a total volume of 2,500 μl.

### RT-qPCR assays for mRNA expression analyses

The TRIzol (Invitrogen, California, USA) method was used for total mRNA extraction and purification, and cDNA was synthesized from 2 μg of total RNA using the ImProm-II Reverse Transcription System (Promega, Madison Wisconsin, USA). mRNA expression detection was performed using a Probe Master kit (Roche, Germany) and hydrolysis probes (Universal Probe Library “UPL”, Roche, Germany) on a LightCycler® 480 Real-Time PCR System (Roche, Germany) following the manufacturer's instructions. mRNA expression levels were normalized to expression of the endogenous gene GAPDH, and expression analyses were performed using the ΔΔT method.

Oligonucleotide sequences and UPL probes (Supplementary Table III) were applied under the following amplification conditions: initial denaturation at 95°C for 5 min and 40 cycles of denaturation at 95°C for 10 seconds, annealing at 60°C for 17 seconds and extension at 72°C for 1 seconds. Oligonucleotides were designed using the ProbeFinder program version 2.51 (Roche, Germany) and synthesized by SIGMA-ALDRICH (USA).

### Western blot assays

Total proteins were extracted from cultured cells and purified using the TRIZOL method (Invitrogen), suspended in 5% SDS with protease inhibitors (cOmplete Mini, Roche, Indianapolis, IN, USA) and quantified using a commercial protein assay kit (Bio-Rad DC). Next, 30 μg of total protein was resolved by vertical electrophoresis in 12% acrylamide gels and then transferred to nitrocellulose membranes using Trans-Blot-Turbo equipment/Transfer System Pierce G2 (Thermo Fisher Scientific, Foster City, CA, USA). The membranes were blocked at room temperature for 2 hours with 5% low-fat milk in 1X PBS/0.1% Tween-20 and incubated overnight with antibodies against GLI1 (Rockland, 100-401-223, Pottstown, PA USA; and Abcam AB134906, Cambridge Science Park, Cambridge, U.K.) (1:3,000), MEOX2 Sc-81971 (1:1,500), and GAPDH Sc-25778 (1:3,000) from Santa Cruz Biotechnology (Dallas, TX, USA). The membranes were washed with 1X PBS supplemented with 0.1% Tween-20 at room temperature and then incubated for 2 hours with a secondary antibody (anti-mouse HRP/anti-rabbit HRP; 1:10,000) at room temperature, followed by washing with 1X PBS supplemented with 0.1% Tween-20 at room temperature. The membranes were incubated with Super Signal West Femto Maximum Sensitivity Substrate (Thermo Fisher Scientific, Fermentas Life Science, Foster City, CA, USA), and a C-Digit Scanner was used for image acquisition (LI-COR, Lincoln, NE, USA). The change ratio was calculated using Image Studio Software (version 4.0.21).

### Cellular viability (Cytotoxicity) assays

Cellular viability analyses were performed in 96-well plates using 3-(4,5-dimethylthiazol-2-yl)-5-(3-carboxymethoxyphenyl)-2-(4-sulphophenyl)-2H-tetrazolium (MTS) (Promega, Madison, WI, USA) in the presence or absence of cisplatinum. Briefly, 96-well plates were seeded with 2,000 to 3,000 cells in 100 μl of RPMI-1640 culture medium for 48 hours and subsequently incubated with 20 μl of MTS (5 mg/ml) for 3 hours to be read later at 490 nm; cell viability was calculated using the following formula: cell viability = (O.D. of the sample/Control O.D.) × 100. Similarly, drug resistance curves were constructed to evaluate dose responses using serial dilutions of cisplatinum.

### Cellular scratch (Migration) assays

Wounding (approximately 800 microns was carried out on cells 8 hours after monolayer cultures were established using 2×10^5^ cells per well (24-well plates)). The cells were deprived of Fetal Bovine serum (FBS) for the remainder of the 48-hours experiment; then, cultures were washed 3 times with 1X PBS. Photographs (40x) were taken at 0, 24, 36 and 48 hours post-wounding. The wound area was quantified using ImageJ, employing the “Duplicate” “Process”, “Binary” and “Make binary” applications to transform pixels into binary code. Finally, adjustments to “Scale Set” and “Analyze Particles” facilitated quantitative representation of the cell migration index expressed as a percentage (%) with respect to the wound (scratch) at time 0 hours.

### Cellular transwell (Migration) assays

Six-well plates were seeded in triplicate at a density of 3×10^5^ cells per well. Following overnight adherence, cells were transfected with corresponding siRNAs for 6 hours. Subsequently, 25×10^3^ transfected cells were transferred into upper transwell chambers in 24-well plates, while the bottom chambers contained RPMI-1640 medium supplemented with 10% FBS. The total culture time was 48 hours at 37°C and 5% CO_2_. Then, cells that adhered below the transwell inserts were carefully fixed using 70% methanol, stained with 0.1% crystal violet, washed with running water, and mounted on slides using entellan. Final images were taken with light field microscope at a total magnification of 100X.

### Clonogenic assays

Six-well plates were seeded at a density of 3×10^5^ cells per well. After overnight adherence, cells were transfected with corresponding siRNAs. The cells were then transferred in triplicate into 6-well plates at a density of 1×10^4^ per well and into 12-well plates at a density of 5×10^3^ per well. Cells were cultured for 6 to 21 days at 37°C and 5% CO_2_, followed by fixation with methanol and acetic acid (3:1 v/v), washing in running water, and staining with 0.5% crystal violet. Colony growth was quantified using the ImageJ Colony Number plugin tool [57].

### Immunohistochemical assays using histopathological samples

Immunohistochemical analyses were performed on FFPE-LT tissue samples, which were used to construct tissue microarrays (TMAs). Lung cancer specimens were blindly selected by a pathologist who identified triplicate lung tumor regions characterized by high cellularity for each lung tumor. The arrays were constructed using the Chemicon Advanced Tissue Arrayer (ATA100) with a 0.5-mm needle. We used 1-2 μg of specific anti-MEOX2 (Santa Cruz Biotechnology, Dallas, Texas, USA) and anti-GLI-1 antibodies (Rockland, Pottstown, PA USA). UltraView Universal DAB and UltraView Universal Alkaline Phosphatase Red detection kits were used in conjunction with a Ventana System BenchMark GX (Ventana Medical System Inc., Tucson, AZ, USA). The slides were dehydrated, cleared in xylol and mounted in synthetic resin. A Leica DM750 transmitted light microscope (Wetzlar, Germany) was used to obtain photomicrographs at 200X and 400X magnification.

The image analysis software program ImageJ and the ImmunoRatio Plugin (Version 1.42q), developed by Jorma Isola and Vilppu Tuominen at the Institute of Biomedical Technology, University of Tampere (http://153.1.200.58:8080/immunoratio/), were used to quantitatively analyze the percentage corresponding to positive nuclear area (average) on histopathological slides (3 fields for each slide sample) at 200X and 400X. In addition, Aperio ePathology ImageScope Software version 11.0.2.725 (Leica Biosystems, USA) and the Intensity Index based on the Algorithm Positive Pixel Count 2004-08-11 (Version 8.100) were used to categorize positive pixel counts into three Intensity Bins, expressed as the Number of Strong Positive Reactions Index (described as Nsr=Nsp/Nwp+Np+Nsp). All data were statistically analyzed to obtain significant differences among the NSCLC patient groups.

### Statistical analyses

*T*-tests, one-way ANOVA, Tukey’s, Dunn's and Mann-Whitney´s multiple comparison tests were performed to analyze the differences between patient groups; *p* values ≤ 0.05 were considered statistically significant. Overall survival analyses were calculated using the Kaplan-Meier method, including comparisons among subgroups with the Breslow-Wilcoxon and Log Rank “Mantel-Cox” tests; *p* values ≤ 0.01, 0.005 and 0.0025 were considered statistically significant. Adjustments for potential confounders were calculated using a multivariate Cox regression model, and the relative risk (RR) ratio was calculated with a corresponding 95% C.I. as a measure of association. All statistical analyses were carried out using the software programs GraphPad (Mac-OS X, Version 5.0) and IBM-SPSS Statistics (Windows, version 20).

## SUPPLEMENTARY FIGURES AND TABLES






