# Supplementary material for: Epigenomic study identifies a novel mesenchyme homeobox2-GLI1 transcription axis involved in cancer drug resistance, overall survival and therapy prognosis in lung cancer patients
Source: Oncotarget. 2017 May 9;8(40):67056–81. doi: 10.18632/oncotarget.17715 (PMC5620156; doi:10.18632/oncotarget.17715)
Supplement: Supplementary file 1 [file oncotarget-08-67056-s001.pdf]

# Epigenomic study identifies a novel mesenchyme homeobox2-GLI1 transcription axis involved in cancer drug resistance, overall survival and therapy prognosis in lung cancer patients

## SUPPLEMENTARY MATERIALS

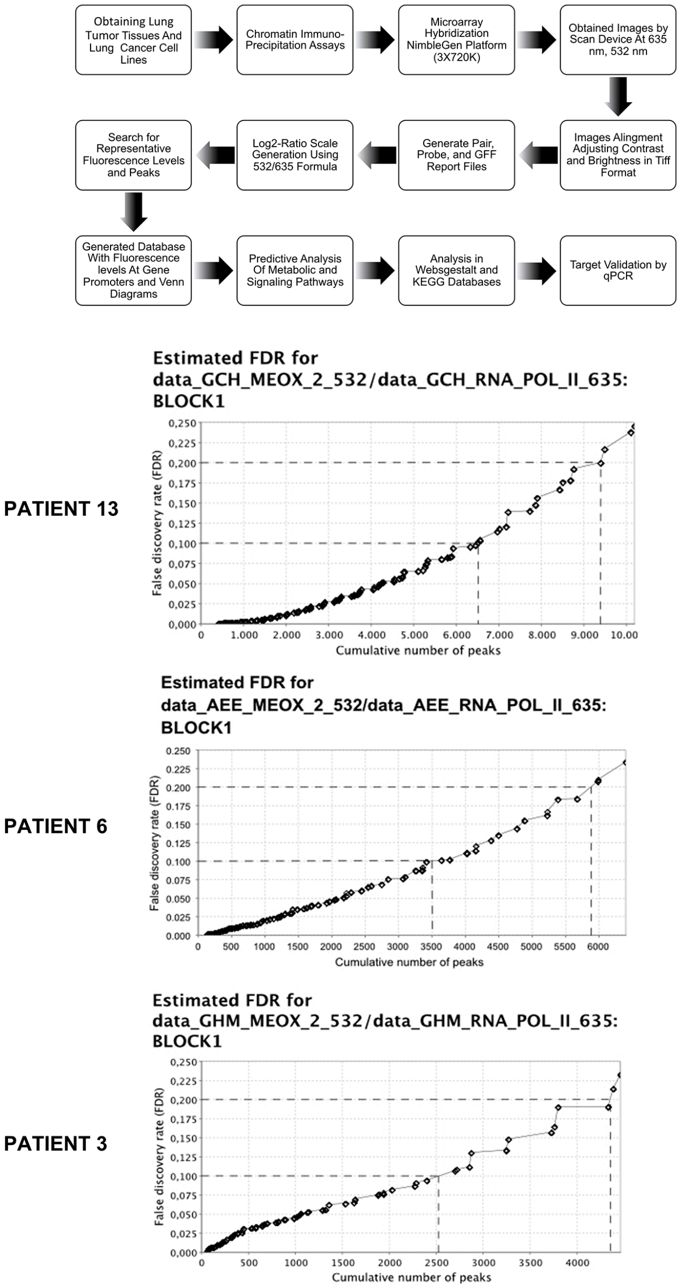


**Supplementary Figure 1: Bioinformatics-data and epigenome analyses obtained from the human solid lung carcinomas.** **(A)** A Pipeline scheme which is describing the epigenome data curation and filtering steps followed for to the identification of gene promoter sequences, which includes to GLI-1 gene. **(B)** Detection of the fluorescence peaks (MEOX2-gene promoter targets) using statistical analyses with FDR≤0.1 and FDR≤0.2; peaks of fluorescence have been highlighted with dotted lines for each lung carcinoma patient.

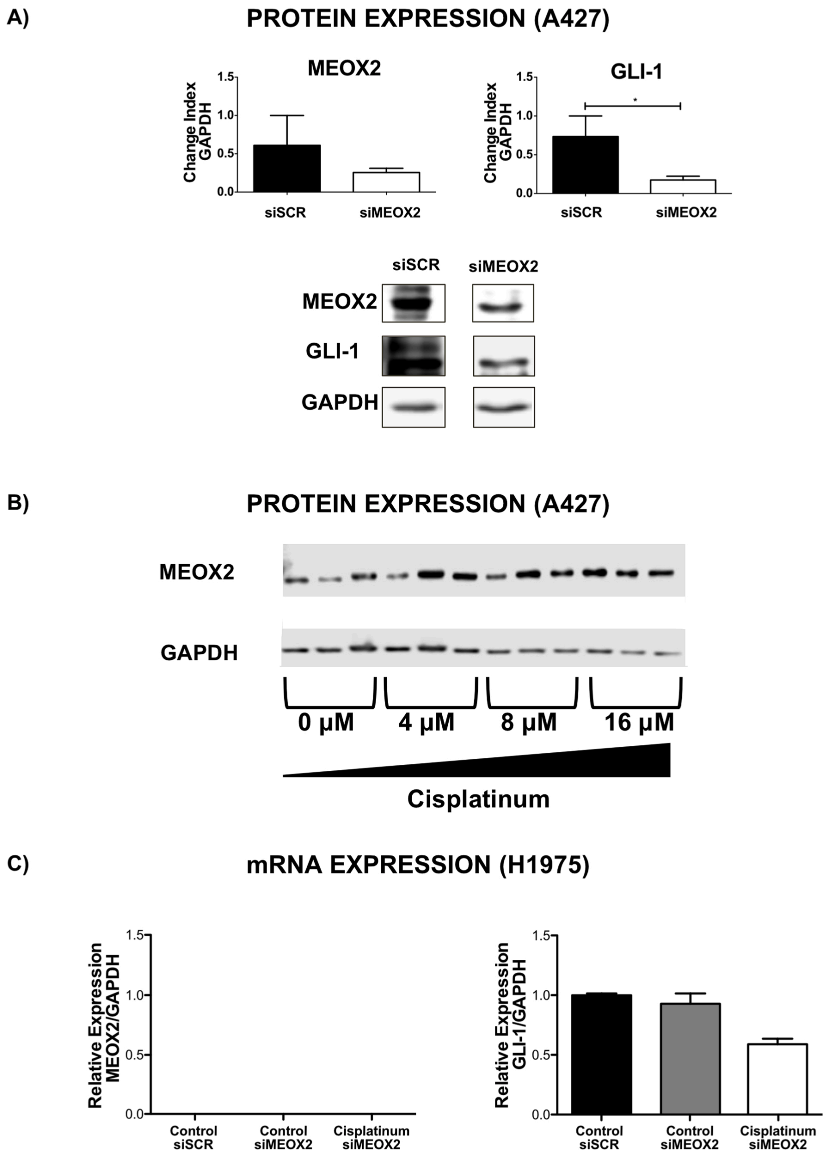


**Supplementary Figure 2: Protein expression analysis in the absence or presence of cisplatinum and genetic silencing siRNA-assays in lung adenocarcinoma cells. (A)** Western blot statistic analyses of the MEOX2 and GLI-1 protein expression using control scramble siRNAs (SCR) and a specific anti-MEOX2 siRNA cocktail (siMEOX2) by *in vitro* genetic silencing assays in A427 lung adenocarcinoma cells. Quantitative densitometry were done at *$p \leq 0.05$ determined by One-Way ANOVA with Dunnett's and Tukey's Multiple Comparison Tests. **(B)** MEOX2 protein inducible expression detected in a cisplatinum-dose dependent manner using a range of IC:6.25 (4 µM), IC:12.5 (8 µM) and IC:25 (16 µM) in A427 lung adenocarcinoma cells. **(C)** mRNA expression analysis in the H1975 lung adenocarcinoma cell line, in the absence or presence of the cisplatinum-based treatment, and using negative control scramble siRNAs (SCR) and specific anti-MEOX2 (siMEOX2) by *in vitro* genetic silencing assays, are shown results from two representative biological experiments by triplicate.

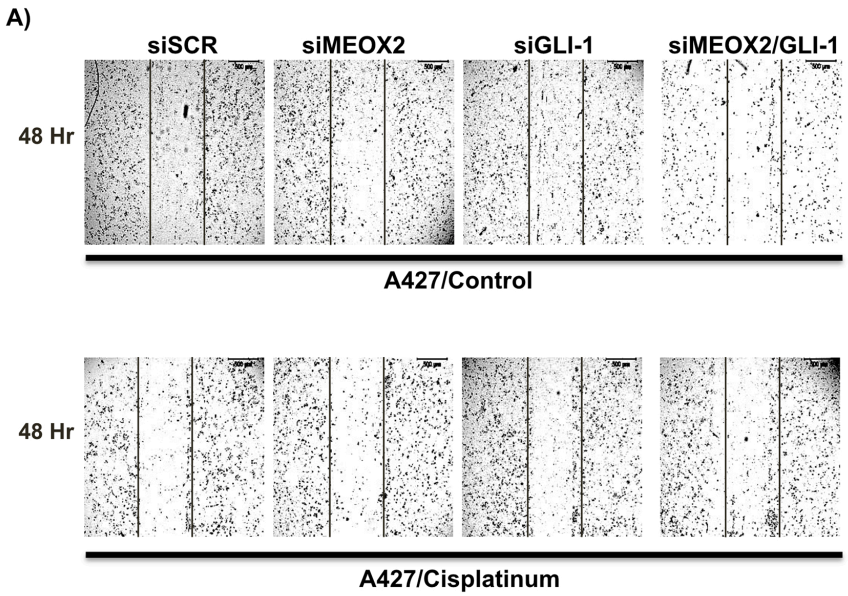


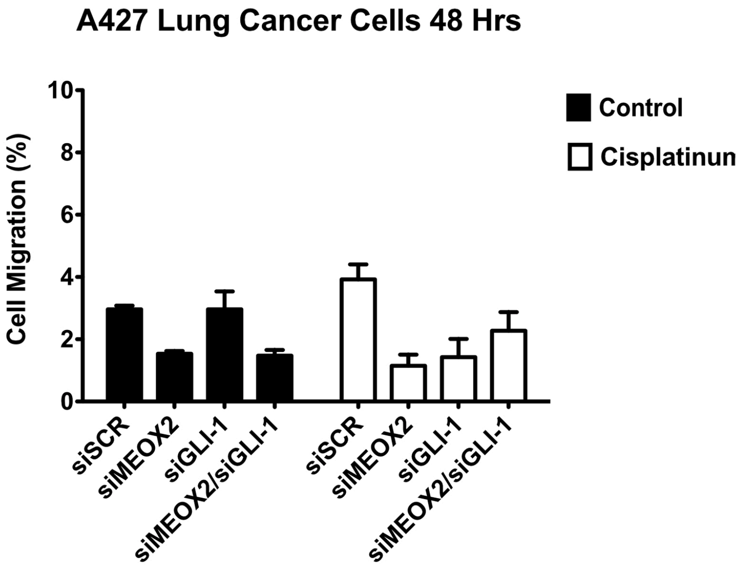


**Supplementary Figure 3: Cellular migration capacity occurs in A MEOX2 and GLI-1 dependent manner in lung adenocarcinoma cells A427 and A549. (A)** Lung cancer cells A427 had a diminished migration capacity by use of individual or mixed anti-MEOX2 and anti-GLI-1 siRNAs in genetic silencing assays, with a non-significant MEOX2 and/or GLI-1 protein dependent functionality using an IC:12.5 (8 µM) cisplatinum-based treatment.

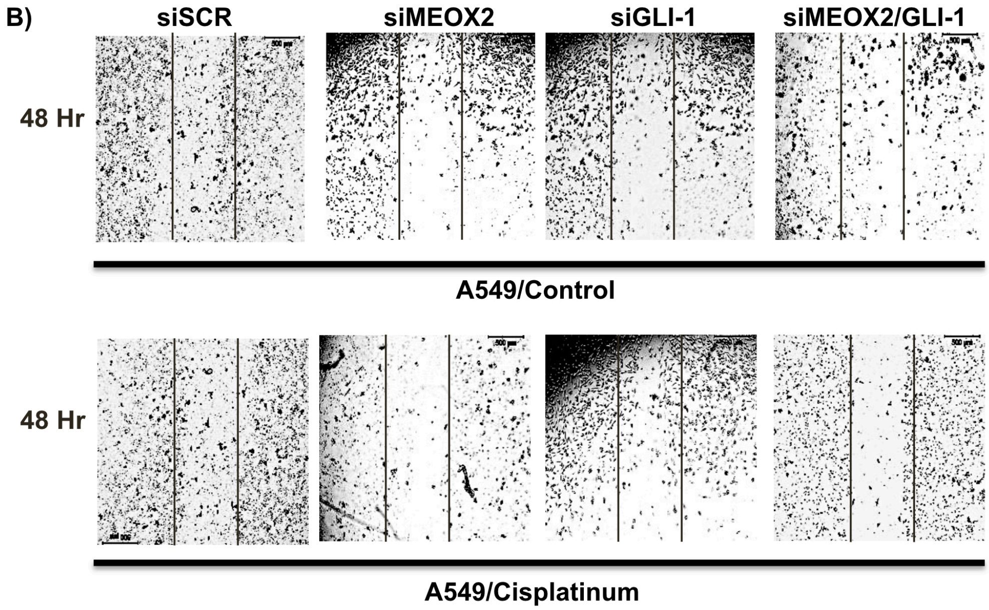


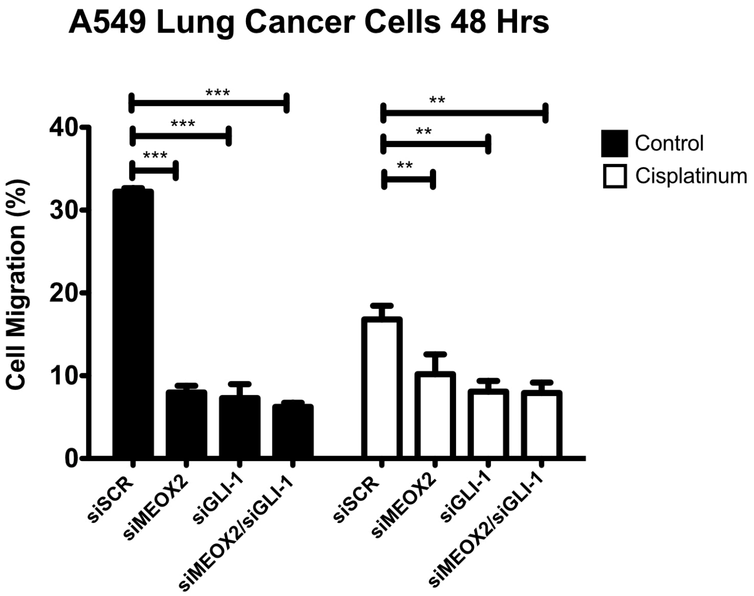


**Supplementary Figure 3: (*Continued* )Cellular migration capacity occurs in A MEOX2 and GLI-1 dependent manner in lung adenocarcinoma cells A427 and A549 (B)** Lung cancer cells A549 had a significant diminished migration capacity, defined by individual and/or mixed anti-MEOX2 and anti-GLI-1 siRNAs, with significant differences for MEOX2 and/or GLI-1 proteins detected using an IC:12.5 cisplatinum-based treatment (**$p \leq 0.01$, and ***$p \leq 0.001$). Statistically significant differences using an one-way ANOVA and Bonferroni's Multiple Comparison Test.

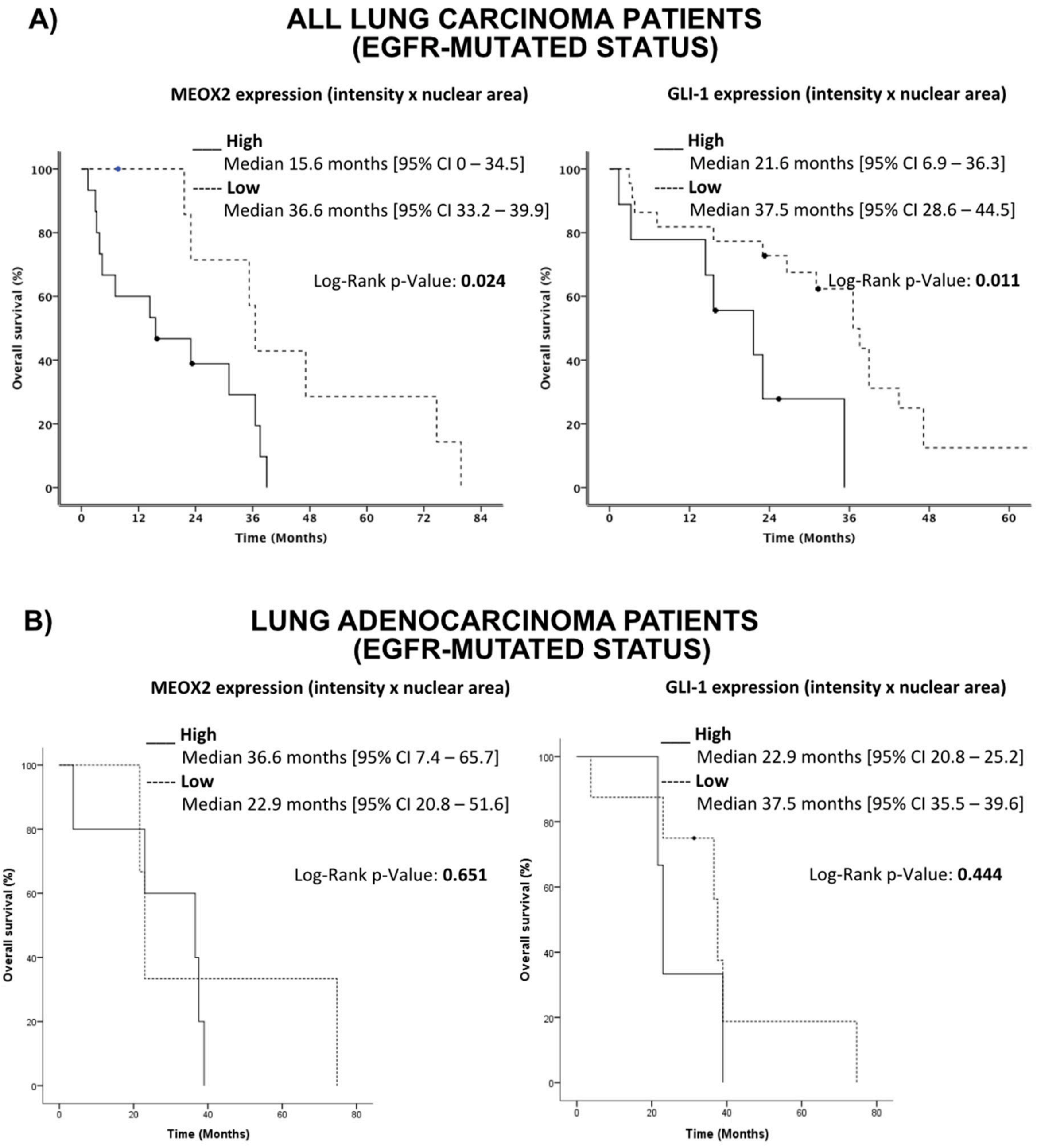


**Supplementary Figure 4: Quantitative (Intensity x Nuclear area index) analysis of the MEOX2-GLI1 expression patterns associated with overall survival in EGFR-mutated lung carcinoma patients. (B)** Kaplan-Maier Overall survival curve analysis studying lung adenocarcinoma patients with EGFR-mutated status with Low or High MEOX2-GLI1 axis expression level, global survival was expressed as Months Accumulated, under cancer-drug cisplatinum-based therapy and/or under combined TKI-based treatment within an EGFR-mutated status, with MEOX2 and GLI-1 expression (Log Rank "Mantel-Cox" $p$=0.651 or 0.444, respectively).

**Supplementary Table I: MEOX2 Targeted Gene Promoter Sequences Obtained By Use Log2 Ratio, Using FDR 0.1 and FDR 0.2 (See Material and Methods) in Lung Adenocarcinoma Patients. Orange color, shows to GLI-1 Gene Promoter Belonging to the Sonic Hedgehog Cell-Signaling Pathway.**

**See Supplementary File 1.**

**Supplementary Table II: Oligonucleotide Sequences Used In Gene Promoter Amplification Analyses, Including Genetic Regions Analyzed on GLI-1 Gene Promoter.**

**See Supplementary File 2.**

**Supplementary Table III: Universal probe library "UPL" hydrolysis probes numbers (Roche, Germany) and oligonucleotide sequences used for RT-qPCR assays and mRNA expression level analyses**

| GENE | UPL PROBE | SEQUENCE (5´- 3´) | SIZE | TM EXP |
|---|---|---|---|---|
| **MEOX2** | **56** | AGAGGAAAAGCGACAGCTCA<br>AAGTTCTCTGATTTGCTCTTTGGT | 110 | 60 |
| **GLI-1** | **1** | CCAGGAATTTGACTCCCAAG<br>GGCTTTGAAGGGCCTCAG | 124 | 60 |
| **GAPDH** | **60** | AGCCACATCGCTCAGACAC<br>GCCCAATACGACCAAATCC | 66 | 60 |
