# Supplementary material for: Epigenomic study identifies a novel mesenchyme homeobox2-GLI1 transcription axis involved in cancer drug resistance, overall survival and therapy prognosis in lung cancer patients
Source: Oncotarget. 2017 May 9;8(40):67056–81. doi: 10.18632/oncotarget.17715 (PMC5620156; doi:10.18632/oncotarget.17715)
Supplement: Supplementary file 2 [file oncotarget-08-67056-s002.docx]

**SUPPLEMENTARY TABLE I.**

| *PEAK_*  *ID FDR* | *CHR* | *PEAK*  *START* | *PEAK*  *END* | *PEAK*  *SCORE* | *PEAK*  *FDR* | *FEATURE*  *STRAND* | *ACCESS* | *DESCRIPTION* |
| --- | --- | --- | --- | --- | --- | --- | --- | --- |
| FDR 0.1 | | | | | | | | |
| 7382 | chr15 | 56096034 | 56096740 | 0.575148 | 0.09818 | - | NM_170697 | ALDH1A2 |
| 1291 | chr20 | 11843414 | 11844612 | 0.570716 | 0.02392 | + | NM_014962 | BTBD3 |
| 7629 | chrX | 33139418 | 33139811 | 0.559561 | 0.09625 | - | NM_004006 | DMD |
| 905 | chrX | 135054343 | 135055220 | 0.606675 | 0.0393 | + | NM_001449 | FHL1 |
| 1023 | chr12 | 56135001 | 56135472 | 0.596296 | 0.07544 | + | NM_005269 | GLI-1 |
| 1353 | chr3 | 11150683 | 11151068 | 0.563425 | 0.07544 | + | NM_001098213 | HRH1 |
| 3098 | chr6 | 119711930 | 119712915 | 0.422957 | 0.0462 | - | NM_005907 | MAN1A1 |
| 7208 | chr20 | 33276879 | 33277332 | 0.585966 | 0.08287 | + | NM_006690 | MMP24 |
| 2583 | chr4 | 71788504 | 71788888 | 0.878884 | 0.01453 | + | NM_001130709 | RUFY3 |
| 4816 | chr2 | 159530737 | 159531771 | 0.705989 | 0.04685 | + | NM_033394 | TANC1 |
| 726 | chr5 | 170667431 | 170667978 | 0.631767 | 0.03231 | + | NM_021025 | TLX3 |
| 2331 | chr8 | 87423914 | 87424506 | 0.567401 | 0.03991 | + | NM_007013 | WWP1 |
| 3276 | chr10 | 31644444 | 31645140 | 0.448939 | 0.06282 | + | NR_024285 | ZEB1 |
| FDR 0.2 | | | | | | | | |
| 4213 | chr1 | 177465419 | 177465885 | 0.479067 | 0.18459 | - | NM_007314 | ABL2 |
| 1061 | chr1 | 177380510 | 177381312 | 0.589386 | 0.04324 | - | NM_005158 | ACSL3 |
| 3587 | chr1 | 177378554 | 177379039 | 0.795589 | 0.0535 | - | NM_001136000 | ALDH1A2 |
| 1865 | chr2 | 223433636 | 223434621 | 0.633109 | 0.00752 | + | NM_004457 | ANKRD50 |
| 3863 | chr2 | 223432452 | 223432818 | 0.427995 | 0.191 | + | NM_004457 | ANKRD53 |
| 6668 | chr2 | 223432756 | 223433449 | 0.609718 | 0.08287 | + | NM_203372 | AP1S2 |
| 177 | chr15 | 56144613 | 56145074 | 0.857094 | 0.01728 | - | NM_170696 | ARRDC4 |
| 1057 | chr15 | 56094606 | 56095478 | 0.590198 | 0.07544 | - | NM_170697 | BTBD3 |
| 7382 | chr15 | 56096034 | 56096740 | 0.575148 | 0.09818 | - | NM_170697 | C20orf111 |
| 1698 | chr4 | 125852791 | 125853754 | 0.646305 | 0.00435 | - | NM_020337 | C5orf30 |
| 1663 | chr4 | 125850590 | 125850860 | 0.543137 | 0.14854 | - | NM_020337 | CASC2 |
| 2586 | chr4 | 125851953 | 125852296 | 0.878884 | 0.01453 | - | NM_020337 | CCM2 |
| 3927 | chr2 | 71058774 | 71059342 | 0.497442 | 0.16211 | + | NM_001115116 | CEP170 |
| 2566 | chr2 | 71056003 | 71056572 | 0.485061 | 0.13397 | + | NM_001115116 | CIZ1 |
| 3085 | chr2 | 71057043 | 71057397 | 0.834351 | 0.03487 | + | NM_001115116 | CREM |
| 623 | chrX | 15782540 | 15783131 | 0.759303 | 0.02314 | - | NM_003916 | DACT1 |
| 3192 | chrX | 15785598 | 15786071 | 0.455006 | 0.1072 | - | NM_003916 | DAZ3 |
| 1783 | chrX | 15783390 | 15784087 | 0.985893 | 0.00328 | - | NM_003916 | DMD |
| 905 | chr15 | 96304847 | 96305518 | 0.721764 | 0.04397 | + | NM_183376 | DTNA |
| 3471 | chr15 | 96304971 | 96305416 | 0.442648 | 0.191 | + | NM_183376 | DYRK1A |
| 1547 | chr15 | 96303395 | 96304510 | 1.022486 | 0.0088 | + | NM_183376 | EHBP1 |
| 399 | chr20 | 11819626 | 11820106 | 0.795783 | 0.03641 | + | NM_181443 | EN2 |
| 1291 | chr20 | 11843414 | 11844612 | 0.570716 | 0.02392 | + | NM_014962 | ERBB2IP |
| 3841 | chr20 | 11846301 | 11847415 | 0.771008 | 0.04345 | + | NM_014962 | F3 |
| 286 | chr20 | 42272275 | 42272616 | 0.819189 | 0.03022 | - | NM_016470 | FBXO33 |
| 3617 | chr20 | 42275193 | 42275570 | 0.434831 | 0.15746 | - | NM_016470 | FHL1 |
| 1223 | chr20 | 42273305 | 42273886 | 1.079411 | 0.00154 | - | NM_016470 | FLJ32063 |
| 2025 | chr5 | 102622264 | 102622950 | 0.618241 | 0.07709 | + | NM_033211 | FOXK1 |
| 3020 | chr5 | 102621123 | 102621603 | 0.459467 | 0.15746 | + | NM_033211 | FOXP1 |
| 6375 | chr5 | 102621428 | 102622020 | 0.622095 | 0.11495 | + | NM_033211 | FSTL1 |
| 2155 | chr10 | 119796280 | 119796651 | 0.610494 | 0.08776 | + | NR_026941 | FYN |
| 2735 | chr10 | 119794184 | 119794527 | 0.476998 | 0.13397 | + | NR_026939 | FZD1 |
| 5844 | chr10 | 119794891 | 119795394 | 0.650177 | 0.09625 | + | NR_026941 | GAB2 |
| 220 | chr7 | 45005973 | 45006774 | 0.837568 | 0.02188 | + | NM_031443 | GLI-1 |
| 2769 | chr7 | 45030725 | 45031217 | 0.475841 | 0.13397 | + | NM_001029835 | GTPBP5 |
| 4226 | chr7 | 45005350 | 45005687 | 0.745808 | 0.0535 | + | NM_031443 | HRH1 |
| 4219 | chr1 | 241484640 | 241485328 | 0.479067 | 0.18459 | - | NM_014812 | INTS6 |
| 1420 | chr1 | 241486931 | 241487302 | 0.559916 | 0.08721 | - | NM_001042405 | ITGA1 |
| 6261 | chr1 | 241486327 | 241486888 | 0.629841 | 0.14039 | - | NM_001042404 | KIAA0146 |
| 233 | chr9 | 130008610 | 130009097 | 0.836373 | 0.02314 | - | NM_001131015 | KIF2A |
| 1909 | chr9 | 130005567 | 130006031 | 0.52188 | 0.08721 | - | NM_012127 | LIF |
| 7432 | chr9 | 130007226 | 130007641 | 0.573205 | 0.16712 | - | NM_001131015 | MAGIX |
| 4761 | chr10 | 35455884 | 35456661 | 0.429607 | 0.10251 | + | NM_183060 | MAN1A1 |
| 655 | chr10 | 35466791 | 35467286 | 0.64535 | 0.045 | + | NM_183012 | MDGA1 |
| 375 | chr10 | 35523990 | 35525085 | 1.362276 | 0 | + | NM_182721 | MMP24 |
| 2876 | chr14 | 58174167 | 58174656 | 0.559657 | 0.11139 | + | NM_016651 | NBEA |
| 1687 | chr14 | 58172058 | 58172634 | 0.541994 | 0.05263 | + | NM_001079520 | NKX2-5 |
| 5727 | chr14 | 58173348 | 58173822 | 0.653892 | 0.11495 | + | NM_001079520 | NKX3-2 |
| 4176 | chrY | 23775350 | 23775929 | 0.480305 | 0.05867 | + | NM_020364 | OGFR |
| 738 | chrY | 25369565 | 25371494 | 0.630152 | 0 | - | NM_020364 | PDE4B |
| 8405 | chrY | 25370601 | 25371099 | 0.458075 | 0.16712 | - | NM_020364 | PELO |
| 1172 | chrX | 31194670 | 31195139 | 0.696028 | 0.04148 | - | NM_004016 | PORCN |
| 592 | chrX | 32949427 | 32950108 | 0.657231 | 0.00702 | - | NM_004007 | PPP1R3F |
| 7629 | chrX | 33139418 | 33139811 | 0.559561 | 0.09625 | - | NM_004006 | PRDM13 |
| 5002 | chr18 | 30327531 | 30328100 | 0.402296 | 0.11483 | + | NM_032979 | PRKG1 |
| 517 | chr18 | 30425505 | 30426050 | 0.677605 | 0.0393 | + | NM_001128175 | PRKRA |
| 7851 | chr18 | 30426331 | 30427134 | 0.53307 | 0.09818 | + | NM_001128175 | PVRL3 |
| 1297 | chr21 | 37661955 | 37662407 | 0.6795 | 0.05137 | + | NM_101395 | RGS3 |
| 2899 | chr21 | 37712170 | 37712825 | 0.468277 | 0.13397 | + | NM_130436 | RUFY3 |
| 2321 | chr21 | 37659072 | 37659627 | 0.90875 | 0.01453 | + | NM_101395 | SATB1 |
| 2128 | chr2 | 62786158 | 62786849 | 0.610498 | 0.00968 | + | NM_015252 | SDCCAG8 |
| 3126 | chr2 | 62752061 | 62752534 | 0.456528 | 0.15746 | + | NM_001142615 | SEC24B |
| 3493 | chr2 | 62785538 | 62786021 | 0.802261 | 0.04345 | + | NM_015252 | SORBS2 |
| 3451 | chr7 | 154942384 | 154944409 | 0.52065 | 0 | + | NM_001427 | SPTBN1 |
| 4035 | chr7 | 154941033 | 154941393 | 0.419859 | 0.191 | + | NM_001427 | TANC1 |
| 7069 | chr7 | 154941968 | 154942317 | 0.590432 | 0.14039 | + | NM_001427 | TBX20 |
| 1724 | chr5 | 65257350 | 65257793 | 0.640321 | 0.06909 | + | NM_018695 | TLX3 |
| 3686 | chr5 | 65257456 | 65258001 | 0.43075 | 0.191 | + | NM_001006600 | TTC3 |
| 1533 | chr5 | 65258487 | 65258947 | 1.026457 | 0.00758 | + | NM_001006600 | WASF3 |
| 825 | chr1 | 94779784 | 94780263 | 0.730007 | 0.04397 | - | NM_001993 | WASL |
| 2275 | chr1 | 94782384 | 94782977 | 0.500978 | 0.13397 | - | NM_001993 | WDFY3 |
| 4512 | chr1 | 94782096 | 94782977 | 0.72929 | 0.08081 | - | NM_001993 | WWP1 |
| 5348 | chr14 | 38970780 | 38971537 | 0.35818 | 0.07914 | - | NM_203301 | YTHDF1 |
| 2595 | chr14 | 38973318 | 38973669 | 0.484942 | 0.13397 | - | NM_203301 | ZEB1 |
| 4086 | chr14 | 38972016 | 38972421 | 0.751976 | 0.06601 | - | NM_203301 | ZFHX3 |
| 777 | chrX | 135057775 | 135058236 | 0.738211 | 0.03022 | + | NM_001159700 | ZNF135 |
| 905 | chrX | 135054343 | 135055220 | 0.606675 | 0.0393 | + | NM_001449 | ZNF330 |
| 1283 | chrX | 135103799 | 135104382 | 1.065831 | 0.00168 | + | NM_001159701 | ZNF664 |
