# Supplementary material for: Epigenomic study identifies a novel mesenchyme homeobox2-GLI1 transcription axis involved in cancer drug resistance, overall survival and therapy prognosis in lung cancer patients
Source: Oncotarget. 2017 May 9;8(40):67056–81. doi: 10.18632/oncotarget.17715 (PMC5620156; doi:10.18632/oncotarget.17715)
Supplement: Supplementary file 3 [file oncotarget-08-67056-s003.docx]

**SUPPLEMENTARY TABLE II.**

| GENE (REGION) | SEQUENCE (5´- 3´) | POSITION | SIZE | TM EXP |
| --- | --- | --- | --- | --- |
| GLI-1 (7) | AGGCCGTGTGACATGTGATT  GACAGAGCGAGACTCCGTCT | -2192 to -2009 | 183 | 55 |
| GLI1 (6) | TCGGACTCCTGACTTGAGGT  TCTTCTCCCCACCCAGTTCT | -1830 to -1673 | 157 | 55 |
| GLI1 (5-4) | CCAGCCTGGGCAAATAGTGA  TCAGAGACCCAGCTCAGTCA | -1541 to -1375 | 166 | 55 |
| GLI1 (3) | CCCTCCAGAACTTCGAGACG  GGCTCTGGAAGAAGGTGAGG | -822 to -665 | 157 | 55 |
| GLI1 (2) | TTCCATCCAAAGGGTGAGGC  CCCCGACAACCAGATTGAGG | -612 to -457 | 155 | 55 |
| GLI1 (1) | AAAAAATTTAGTCGTTTCGTTTGA  TTATTAAAACGCTACCTCCGAA | -301 to -109 | 193 | 55 |
|  |  |  |  |  |
